# Novel integrated approach modeling proanthocyanidins and bacteriophages to combat multidrug *Salmonella* Typhimurium in challenged broilers

**DOI:** 10.3389/fvets.2025.1694544

**Published:** 2025-11-26

**Authors:** Hanan S. Al-Khalaifah, Doaa Ibrahim, Ahmed Abdelfattah-Hassan, Dina Ibrahim, Alaaeldin Mohamed Saad, Mai. F. Saad, Sara M. El-Badry, Reham A. Elbhnsawy, Asmaa A. Azouz, Mayada Mahmoud, Sherief M. Abdel-Raheem, Hesham Ismail, Rania M. S. El-Malt, Marwa I. Abd El-Hamid

**Affiliations:** 1 Environment and Life Sciences Research Center, Kuwait Institute for Scientific Research, Safat, Kuwait; 2 Department of Nutrition and Clinical Nutrition, Faculty of Veterinary Medicine, Zagazig University, Zagazig, Egypt; 3 Department of Veterinary Medicine, College of Applied and Health Sciences, A'Sharqiyah University, Ibra, Oman; 4 Department of Anatomy and Embryology, Faculty of Veterinary Medicine, Zagazig University, Zagazig, Egypt; 5 Bacteriology, Mycology, Immunology Department, Faculty of Veterinary Medicine, Mansoura University, Mansoura, Egypt; 6 Zoonoses Department, Faculty of Veterinary Medicine, Zagazig University, Zagazig, Egypt; 7 Department of Veterinary Public Health, Faculty of Veterinary Medicine, Zagazig University, Zagazig, Egypt; 8 Department of Animal Wealth Development, Veterinary Genetics and Genetic Engineering, Faculty of Veterinary Medicine, Zagazig University, Zagazig, Egypt; 9 Department of Poultry and Rabbit Diseases, Veterinary Teaching Hospital, Faculty of Veterinary Medicine, Mansoura University, Mansoura, Egypt; 10 Department of Pharmacology, Faculty of Veterinary Medicine, Cairo University, Giza, Egypt; 11 Department of Microbiology, Animal Health Research Institute, Zagazig, Sharkia, Egypt; 12 Department of Public Health, College of Veterinary Medicine, King Faisal University, Al Hofuf, Al Ahsa, Saudi Arabia; 13 Department of Microbiology, Animal Health Research Institute (AHRI), Zagazig Branch, Agriculture Research Center (ARC), Zagazig, Egypt; 14 Department of Microbiology, Faculty of Veterinary Medicine, Zagazig University, Zagazig, Egypt

**Keywords:** proanthocyanidins and bacteriophages mixture, growth, immunostimulant, gastrointestinal integrity, antioxidant, anti-virulence, *Salmonella* Typhimurium

## Abstract

The emergence of multidrug bacterial isolates, including *Salmonella* (*S*.) Typhimurium, which primarily spreads to humans through chicken products, is correlated with a rising prevalence of antimicrobial therapy failure. Thus, we performed a comprehensive analysis of the combined impact of *Salmonella* bacteriophage (BP) and grape seed oligomeric pro-anthocyanidins (GSOPs) on growth performance, immune functions, antioxidant capacity, cecal microbiota, gut integrity, and *S*. Typhimurium resistance in challenged broilers. A total of 250 Ross-308 male broiler chicks were offered either a control diet or a diet supplemented with *Salmonella* BP alone at concentrations of 10^9^ PFU/0.1 ml, GSOPs alone at a level of 400 mg/kg diet, and a combination of both *Salmonella* BP and GSOPs, and experimentally infected with multidrug-resistant (MDR) *S*. Typhimurium strain at 14 days of age. Broilers administered BP, GSOPs, and their combination, particularly BP+GSOPs, had enhanced growth performance attributes even following a challenge with *S*. Typhimurium, alongside decreased mortality percentage, which was evidenced by increased expression of *MUC-2*, β-defensin-1, cathelicidins-2, *JAM*-2, occludin, and *CLDN-1* genes, reduced *S*. Typhimurium abundance, and downregulating its virulence-associated genes (*sopE* and *spvC*), alongside restored intestinal histological features. GSOPs+BP fortified group exhibited higher cecal beneficial bacteria counts (*Bacteroides, Firmicutes, Lactobacillus*, and *Bifidobacterium* species), lower cecal harmful bacteria loads (*Escherichia, Enterobacteriaceae*, and *Clostridium* clusters I and IV), decreased serum oxidative markers [H_2_O_2_, reactive oxygen species (ROS), and malondialdehyde (MDA)], and increased serum antioxidant enzymes [superoxide dismutase (SOD), catalase (CAT), and glutathione peroxidase (GSH-Px)]. The incorporation of dietary BP, and GSOPs combination significantly downregulated the intestinal inflammatory regulated genes (*IL-1*β*, IL-6, CCL4, CCL20, COX-2*, and *iNOS*), and *mTOR* gene, and reduced the serum concentrations of LYZ, NO, CRP, and complement C3, alongside increased serum IgG, and IgM levels, and upregulation of autophagy-related genes (*atg5, atg7, atg12, BCLN-1*, and *LC3-II*). The aforementioned beneficial effects of the BP and GSOPs combination support their prospective use in avian nutrition to enhance performance and stimulate immune defense against gastrointestinal illnesses, including *S*. Typhimurium infection.

## 1 Introduction

Consumer interest in poultry-derived food is rising, particularly in developing nations, mostly due to enhancements in growth rates, feed conversion ratios, and the management of enteric microbial infections (1, 2). *Salmonella* is one of the most prevalent pathogens affecting avian illnesses. *Salmonella enterica* serovar Typhimurium is a prevalent enteric pathogenic bacterium that poses significant economic detriment to the chicken industry and is linked to human food-borne illnesses resulting from the ingestion of contaminated chickens (3, 4). *Salmonella* species possesses the capacity to express a multitude of virulence-associated proteins (effector proteins), particularly *Salmonella* outer proteins (Sops), which are encoded by *sop* genes that alter the structure and functioning of gastrointestinal tight junction proteins (TJP). In addition, the *Salmonella* plasmid virulence C (*spvC*) gene is a crucial virulence marker for *Salmonella* Typhimurium, as it is essential for endurance and proliferation within poultry macrophages, which is crucial for the complete manifestation of its virulence (5, 6). The extensive application of antimicrobial agents to control microbial infections, including salmonellosis, and as growth enhancers in animal husbandry has led to the creation of multidrug-resistant (MDR) pathogens in both human and animal (7–11), diminishing the efficacy of infection treatment and elevating the danger of fatality (12–15); thus, researchers are exploring novel alternative antibiotics (16, 17), such as phytochemicals (18–20). *Salmonella* spp. has acquired resistance to numerous antimicrobial agents, leading to MDR strains, which have proliferated in both developed and developing nations (21). *Salmonella* infection in chickens is linked to substantial mortality and morbidity, stunted growth, and poor feed efficiency, resulting in major financial losses within the poultry industry (22, 23). Consequently, it is imperative to establish efficient approaches for managing and mitigating salmonellosis within the poultry sector. Various strategies could be implemented alongside farm biosecurity protocols, as distinct modes of action may yield additive synergistic impacts throughout the meat production chain, hence enhancing the safety of the end product (24), and these strategies encompass bacteriophages (BP) (25), and phytochemicals (26–29).

Bacteriophages (BP), or phage therapy, formerly eclipsed by chemical antimicrobials, is experiencing a resurgence in study as a potential treatment for MDR bacterial illnesses, including salmonellosis (22). Bacteriophages are viruses that selectively lyse particular bacterial species or strains. Their specificity to bacterial hosts, non-toxicity, and availability are important features over other antimicrobial interventions, as lytic BP do not impact the gut microbiota (24); thus, they have been employed as effective instruments for several applications, including enhancing food safety and combating bacterial infections (25). Prior research has shown that BP can significantly suppress inflammation at both the cellular and humoral levels, in addition to upregulating genes that activate cellular metabolism, hence decreasing gastrointestinal permeability (30). Lytic BP could be offered via feed and drinking water (31). Sarrami et al. (31) stated that dietary BP inclusion enhanced the growth performance of *Salmonella* Enteritidis challenged broilers by reducing inflammation in monocytes, optimizing metabolism in gut epithelial cells, decreasing the cecal count of *S*. Enteritidis and coliform bacteria, and improving liver health. Numerous investigations have previously indicated the beneficial effects of BP in diminishing *Salmonella* infection and enhancing the production performance of broilers (22, 25, 32, 33).

To maintain gastrointestinal health and reduce the need for antibiotics for gastrointestinal infections (34, 35), substitutes are required in both veterinary and human medicine, in alignment with contemporary consumer preferences for unmedicated, natural substances (36–41). Phytochemicals, including flavonoids, are generated by plants via primary and secondary metabolism, and they are regarded as natural replacements to antimicrobial agents (42–44). Proanthocyanidins (PAs) are flavonoids extensively studied for their use in both animal and human medicine owing to their diverse therapeutic properties. They demonstrate antioxidant, anti-inflammatory, and anticarcinogenic characteristics, as well as efficacy against several pathogens (45). Moreover, PAs are recognized for their capacity to impede bacterial adherence to cells and various surfaces (46). Their protective influence on the cardiovascular (47) and gastrointestinal (48) systems has also been documented. Grape seed extract is a substantial source of PAs among various vegetables, fruits, and seeds (49). Due to their beneficial effects, PAs may function as replacements for antibiotics by safeguarding the gastrointestinal system from bacterial infections (45, 50, 51). Multiple studies have demonstrated the positive effects of PAs in enhancing the growth performance, antioxidant potential, gut health, and immune status of broilers (50, 52–56).

Prior investigations effectively integrated *Salmonella* BP (22, 25, 33) and anthocyanins (26, 57) to diminish *S*. Typhimurium in challenged broilers and improve the production performance of broiler chickens. Nonetheless, despite the encouraging outcomes of individual applications of the two approaches to mitigate *S*. Typhimurium, the synergistic use of *Salmonella* BP and PAs has yet to be investigated for this bacterium. Consequently, the current investigation aimed to examine, for the first time, the combined impacts of *Salmonella* bacteriophages and grape seed oligomeric pro-anthocyanidins (GSOPs) on growth performance, immunity, antioxidant potential, cecal microbial load, gut integrity, in addition to controlling *S*. Typhimurium colonization, and virulence in broilers challenged with MDR *S*. Typhimurium strain.

## 2 Materials and methods

### 2.1 Ethical approval

The entire study was conducted in accordance with the instructions and authorized specifications provided by the Institutional Animal Care and Use Committee (IACUC), Faculty of Veterinary Medicine, Zagazig University, Egypt, with reference no. ZU-IACUC/2/F/192/2024.

### 2.2 Proanthocyanidins and bacteriophage

The grape seed oligomeric pro-anthocyanidins (GSOPs) used in the present study were procured from Sigma-Aldrich (Darmstadt, Germany; purity ≥98.8%).

The *Salmonella* bacteriophage (φSalmacey3) used in the current study was formerly isolated from sewage water samples, as documented in a prior work by one of the co-authors (58). This BP could infect and eliminate three MDR *Salmonella* serovars, including *S*. Typhimurium, *S*. Enteritidis, and *S*. Kentucky, in addition to *E. coli, S*. Typhi, and *Citrobacter freundii*. Titration of phages was done according to Ref. (59). In brief, phage filtrate was serially diluted, and the diluent was sterile saline (0.9% NaCl). To a 3 ml melted semi-solid agar kept at 45 °C, 100 μl of bacterial overnight culture of the tested bacterial isolate was mixed with 100 μl of phage suspension, shaken, and poured quickly onto solid nutrient agar plates. After the double agar overlay solidification, the plates were incubated overnight at 37 °C. Counting of plaques was done after the incubation, and the titer of the phage expressed as plaque-forming units per ml (PFU/ml) was calculated as follows: Number of plaques × 10 × reciprocal of counted dilution.

### 2.3 Broilers, design of experiments, and feeding regimen

The experiment involved 250 male Ross-308 broiler chicks (1-day-old) acquired from a local commercial hatchery. Upon arrival, the chicks were subjected to a bacteriological analysis of fecal samples and cloacal swabs to confirm the absence of *Salmonella* spp., in accordance with the International Organization for Standardization (ISO) 6,579 guidelines (60). The broilers were weighed individually and randomly allocated into five equal treatment groups, with five replicates per group and ten chicks per replicate. The five experimental groups included two control groups; negative control (C) chicks were administered a basal diet devoid of any additives and were not subjected to any challenge, and infective control (IC) chicks were fed a basal diet without supplements and were challenged at 14 days of age with MDR *S*. Typhimurium strain, and three treatment groups where chicks were offered a basal diet augmented with *Salmonella* BP alone at concentrations of 10^9^ PFU/0.1 ml, GSOPs alone at a level of 400 mg/kg diet and a combination of both *Salmonella* BP and GSOPs and were challenged at 14 days of age with MDR *S*. Typhimurium strain. The GSOPs supplemented diets were administered from the first day of life, while BP was added to the mash diet immediately at the time of feeding at 1, 2, 3, 5, 7, 9-, 11-, 13-, and 15 days post-challenge (dpc). Feed and water were administered *ad libitum* over the 36-day experimental trial. All diets were provided in mash form, and the basal diets for the starter (1–11 days), grower (12–21 d), and finisher (22–36 d) stages were produced in accordance with the nutritional specifications outlined in the Ross Broiler Handbook (61), as depicted in Table 1. The chemical tests of all the feed constituents were performed according to the standard procedure established by the Association of Official Analytical Chemists (AOAC) (62).

**Table 1:** The nutrition levels and ingredients of the basal diet.

**Ingredient, %**	**Starter (1–11 days)**	**Grower (12–21 days)**	**Finisher (22–36 days)**
Soybean oil	2.2	3.2	4.2
Soybean meal, 48	35	31.3	26.3
Yellow corn	58	60.8	64.8
Common salt	0.3	0.3	0.3
Calcium diphasic phosphate	1.5	1.5	1.5
Calcium carbonate	1.2	1.2	1.2
Anti-mycotoxin	0.1	0.1	0.1
Choline chloride	0.2	0.2	0.2
DL-Methionine,99	0.25	0.2	0.2
L-Lysine HCL,78	0.35	0.3	0.3
Premix^*^	0.3	0.3	0.3
**Nutrient composition**			
Methionine, %	0.58	0.52	0.49
Lysine, %	1.45	1.3	1.17
Available P, %	0.53	0.51	0.48
Ca, %	1.07	1.07	1.05
CF, %	2.63	2.56	2.46
EE, %	4.63	5.6	6.74
CP, %	23.01	21.5	19.5
ME, Kcal/Kg	3,106	3,103	3,203

^*^Vitamin premix (per 1 kilogram of diet): menadione, 2.5 mg; biotin, 300 μg; thiamine, 4 mg; folate, 3 mg; cyanocobalamin, 15 μg; pyridoxine, 6 mg; niacin, 50 mg; pantothenate, 12 mg; Zn (sulfate and oxide), 120 mg; Cu (sulfate), 14 mg; Se (selenate), 0.3 mg; Mn (sulfate and oxide), 100 mg; I (iodide), 1.20 mg; Fe (sulfate), 30 mg; riboflavin, 7 mg; retinol, 10.000 IU; tocopherol acetate, 70 mg; cholecalciferol, 6,000 IU.

P, phosphorus; Ca, calcium; CF, crude fiber; EE, ether extract; CP, crude protein; ME, metabolizable energy.

### 2.4 *Salmonella* Typhimurium challenge trial

This experiment employed a coccidia-free *S*. Typhimurium challenge model as earlier documented (63). The MDR *S*. Typhimurium strain utilized in the current research was formerly obtained from the visceral organs of diseased and freshly dead broilers and subsequently identified phenotypically using traditional bacteriological methods, as previously described (64, 65). The strain was obtained from suspensions preserved at −80 °C and cultured in brain heart infusion (BHI) broth (Oxoid, UK) at 37 °C/ 24 h. The resulting culture was then streaked over xylose lysine deoxycholate agar (Oxoid, UK) and incubated at 37 °C/24 h. The challenge inoculum was created by properly diluting the suspension in BHI broth to achieve a final viable cell concentration of 3 × 10^6^ CFU/ml for the oral infection of challenged broilers. At 14 days of age, all experimental broilers were administered the challenge inoculum of *S*. Typhimurium by oral gavage using a syringe equipped with a flexible tube (66). The infection was monitored throughout the trial via re-isolation and identification of the challenging *S*. Typhimurium strain from cecal specimens of euthanized and dead broilers. Clinical symptoms, mortality, and gross lesions were documented immediately following the challenge and continued until the conclusion of the experiment.

### 2.5 Growth performance

The individual body weight (BW) and feed intake (FI) for each group were documented to compute body weight gain (BWG) and feed conversion ratio (FCR) at the conclusion of the starter, grower, and finisher phases. Feed intake of broiler chicks/pen was estimated individually, and FCR was calculated as follows: total feed intake per replicate/total body weight gain per replicate. At the end of the trial, FI, BWG, and FCR were computed for the entire growing period (1–36 d) as earlier outlined (67, 68).

### 2.6 Collection of samples

At 7 days post-challenge (dpc), serum and intestinal specimens were utilized to assess the immunological, biochemical, oxidative, and antioxidant-related biomarkers. At 7 dpc, intestinal and cecal tissues (5/group) were further preserved in RNA Later (Sigma, USA) for analyzing differential gene expressions of intestinal barrier functions, autophagy, inflammation, and S. Typhimurium virulence using quantitative reverse transcription polymerase chain reaction (RT-qPCR) methods. Cecal contents (5/group) were aseptically obtained and utilized for further quantification of S. Typhimurium counts (at 7- and 14-dpc), and cecal microbial counts (at 7-dpc) by quantitative real-time PCR (qPCR) technique. Finally, at the conclusion of the experimental period (36 days of age), the cecal tissues of broilers (5/group) were aseptically collected, rinsed with sterile PBS, and used for histopathological evaluation.

### 2.7 Assessment of broilers' antioxidant capacity

At 7 dpc, serum levels of superoxide dismutase (SOD), catalase (CAT), glutathione peroxidase (GSH-Px), malondialdehyde (MDA), and reactive oxygen species (ROS) were measured using commercially available kits obtained from Nanjing Jiancheng Bioengineering Research Institute (Nanjing, China) as per the manufacturer's guidelines. Additionally, hydrogen peroxide (H_2_O_2_) concentrations were assessed as ml of serum according to previously established protocols (63, 69).

### 2.8 Serum biochemical and immunological analysis

At 7 dpc, the serum concentrations of alanine and aspartate aminotransferases (ALT and AST), creatinine, and urea, as well as the activities of nitric oxide (NO) and lysozyme (LYZ) were evaluated applying commercial analytical kits (Jiancheng Biotechnology Institute™, Nanjing, China) in compliance with the manufacturer's instructions. The serum levels of immune-related indicators, including complement protein C3, immunoglobulin M (IgM), and IgG, were measured using enzyme-linked immunosorbent assay (ELISA) kits (Sigma Aldrich, USA) as per the manufacturer's guidelines at 7 dpc (1). Furthermore, at 7 dpc, the serum concentration of C-reactive protein (CRP) was assessed as previously reported (2).

### 2.9 Quantification of cecal microbiota and *Salmonella* Typhimurium DNA copies by quantitative real-time PCR assay

At 7 and 14 dpc, cecal contents were used for a quantitative real-time PCR assay. A pre-extraction step was conducted for better performance of DNA extraction from the investigated bacteria via mechanical lysis using glass beads and acid washing, followed by vortexing and thermal lysis at 65 °C. DNA extraction was then done via the QIAamp DNA Mini kit (Qiagen, Germany) in accordance with the manufacturer's instructions. Absolute quantification of *S*. Typhimurium (at 7 and 14 dpc), and various cecal microbial populations including *Lactobacillus* spp., *Bifidobacterium* spp., *Bacteroides, Firmicutes, Escherichia, Enterobacteriaceae, Clostridium* clusters I and IV (at 7 dpc) were performed using the qPCR approach, in triplicate, employing the QuantiTect SYBR Green PCR Master Mix (Qiagen, Germany) as per the manufacturer's instructions, and Stratagene MX3005P RT-PCR machine. Table 2 presents the primer sequences for the various examined bacterial DNA. The selected primers showed good standard curve validation data with a square regression coefficient (*R*^2^) of ≥0.992, reaction efficiency of above 92.75%, and limit of detection of −2.29 to −2 (Supplementary Table S1). The extracted DNA from pure bacterial cultures was serially diluted ten times to estimate the qPCR standard calibration curves. The target genomic DNA copies were quantified, and bacterial levels in the cecal content were represented as log_10_ CFU/gram.

**Table 2:** Primers' sequences employed for PCR assays.

**Specificity/target gene**	**Primer sequence (5′-3′)**	**Accession No./reference**
***Salmonella*** **Typhimurium**		
*invA*	F-GTGAAATTATCGCCACGTTCGGGCAA	KF026356
	R- TCATCGCACCGTCAAAGGAACC	
**Cecal microbiota**		
*Enterobacteriaceae*	F-CATTGACGTTACCCGCAGAAGAAGC	(129)
	R-CTCTACGAGACTCAAGCTTGC	
*Firmicutes*	F-GGAGYATGTGGTTTAATTCGAAGCA	(130)
	R-AGCTGACGACAACCATGCAC	
*Escherichia*	F-GTTAATACCTTTGCTCATTGA	
	R-ACCAGGGTATCTAATCCTGT	
*Bacteroidetes*	F-GGARCATGTGGTTTAATTCGATGAT	
	R-AGCTGACGACAACCATGCAG	
*Bifidobacterium* species	F-TCGCGTCYGGTGTGAAAG	
	R-CCACATCCAGCRTCCAC	
*Lactobacillus* species	F-AGCAGTAGGGAATCTTCCA	
	R-CACCGCTACACATGGAG	
*Clostridium* cluster IV	F-GCACAAGCAGTGGAGT	
	R-CTTCCTCCGTTTTGTCAA	
*Clostridium* cluster I	F-TACCHRAGGAGGAAGCCAC	(131)
	R-GTTCTTCCTAATCTCTACGCAT	
**House keeping**		
*GAPDH*	F-GGTGGTGCTAAGCGTGTTA	NM205518
	R-CCCTCCACAATGCCAA	
*rpoD*	F-ACATCGCTAAACGTATCGAA	(63)
	R-GTACTGTTCCAGCAGATAGG	
**Intestinal barrier functions**		
*CLDN-1*	F-GGTGAAGAAGATGCGGATGG	NM_001013611
	R-TCTGGTGTTAACGGGTGTGA	
occludin	F-ACGGCAAAGCCAACATCTAC	XM_031604121.1
	R- ATCCGCCACGTTCTTCAC	
*JAM-2*	F-AGACAG GAACAGGCAGTGCT	XM_031556661.1
	R- TCCAATCCCATTTGA GGCTA	
*MUC-2*	F-AAACAACGGCCATGTTTCAT	NM_001318434
	R- GTGTGACACTGGTGTGCTGA	
β-defensin-1	F-AAACCATTGTCAGCCCTGTG	NM_204993.1
	R-TTCCTAGAGCCTGGGAGGAT	
Cathelicidins-2	F-AGGAGAATGGGGTCATCAGG	NM_001024830.3
	R-GGATCTTTCTCAGGAAGCGG	
**Immune response-related genes**		
*IL-1β*	F-TTCCGGATGTATCTCGAGCA	NC_013670
	R- GTGGATCGTGGTCGTCTTCA	
*IL-6*	F-AGGACGAGATGTGCAAGAAGTTC	NM_205498.1
	R- TTGGGCAGGTTGAGGTTGTT	
*CCL4*	F: GCAGTTGTTCTCGCTCTTC	NM_204720.1
	R: GCGCTCCTTCTTTGTGAT	
*CCL20*	F-AGGCAGCGAAGGAGCAC	NM_204438
	R-GCAGAGAAGCCAAAATCAAAC	
*iNOS*	F-AGGTGGCAGAGAGATGAACGAA	HQ589354.1
	R-AGGAGGCTTTGTGAGGGTGG	
*COX-2*	F-TGTCCTTTCACTGCTTTCCAT	NM_0,011,67718.1
	R-TTCCATTGCTGTGTTTGAGGT	
**Autophagy**		
*mTOR*	F-TGCGGAGTATGTGGAGTT	XM_019108641.1
	R-CATCTCTTTGGTCTCTCTCTGG	
*LC3-II*	F-GGAACAGCATCCAAGCAAGA	NM199604.1
	R-TCAGAAATGGCGGTGGACA	
*BCLN-1*	F-TCTGTTTGATATCATGTCTGG	XM_019068185.1
	R-TAATTCTGGCACTCATTTTCT	
*atg5*	F-ATTGGCGTTTTGTTTGATCTT	XM_019082404.1
	R-TTTGAGTGCATCCGCCTCTTT	
*atg7*	F: ACTGGCAATGCGTGTTTCAG	NM_001030592
	R: CGATGAACCCAAAAGGTCAGA	
*atg12*	F-ACAGTACAGTCACTCGCTCA	XM_019125508.1
	R-AAAACACTCGAAAAGCACACC	
***S***. **Typhimurium virulence**		
*sopE*	F-CGAGTAAAGACCCCGCATAC	(132)
	R-GAGTCGGCATAGCACACTCA	
*spvC*	F-ACTCCTTGCACAACCAAATGCGGA	
	R-TGTCTCTGCATTTCGCCACCATCA	

invA, S. Typhimurium invasion protein A gene; GAPDH, glyceraldehyde 3-phosphate dehydrogenase; rpoD, RNA polymerase sigma factor; sopE, Salmonella outer protein E; spvC, Salmonella plasmid virulence C; LC3-II, microtubule-associated proteins 1A/1B light chain; mTOR, mechanistic target of rapamycin; BCLN-1, beclin-1; atg, autophagy related genes; COX-2, cycloox-ygenase-2; iNOS, inducible nitric oxide synthase; CCL, chemokine C–C motif ligand; IL, interleukin; CLDN-1, claudins-1; JAM-2, junctional adhesion molecule-2; MUC-2, mucin-2.

### 2.10 Gene expression analysis of genes encoding barrier functions, autophagy, inflammation, and *S*. Typhimurium virulence via reverse transcription-quantitative PCR technique

At 7 dpc, broilers' intestinal and cecal samples were utilized to extract and purify total RNA via the QIAamp RNeasy Mini kit (Qiagen GmbH, Germany) as per the manufacturer's directions. The Spectrostar NanoDrop™ 2000 spectrophotometer (Thermo Fisher Scientific Inc., USA) was used to measure the extracted RNA concentration at 260 nm optical density. The absorbance ratio at 260 and 280 nm was then used to confirm the RNA purity. The transcriptional levels of genes related to intestinal barrier functions [mucin-2 (*MUC-2*), claudins-1 (*CLDN-1*), occludin, junctional adhesion molecule (*JAM-2*), cathelicidins-2, and β-defensin-1,], inflammation [interleukin (IL)-1 beta (*IL-1*β), *IL-6*, C-C motif chemokine ligand 4 (*CCL4*), *CCL20*, cyclooxygenase-2 (*COX-2*), and inducible nitric oxide synthase (*iNOS*)], autophagy [autophagy (atg) related gene-5 (*atg5*), *atg7, atg12*, microtubule-associated proteins 1A/1B light chain (*LC3-II*), beclin-1 (*BCLN-1*) and mechanistic target of rapamycin (*mTOR*)], and *S*. Typhimurium virulence [*spvC* and *sopE*] were determined by one-step RT-qPCR amplification technique, in triplicate, utilizing the QuantiTect SYBR Green RT-PCR Kit (Qiagen Germany) on a Stratagene MX3005P machine (Agilent Technologies, Inc., USA) following the protocol's manuals. In order to determine if non-specific amplification products were present or not, a melting curve analysis was conducted after amplification. The appropriate gene-specific primer sets employed for evaluating the expression analysis are listed in Table 2. The transcription levels of glyceraldehyde 3-phosphate dehydrogenase (*GAPDH*) and RNA polymerase sigma factor (*rpoD*) were utilized as internal housekeeping genes for the expression levels of the genes under investigation. Both reference genes were selected based on their lower expression stability values, M, which were evaluated via the GeNorm tool. The 2^−Δ*ΔCt*^ approach was utilized to assess the relative gene expression data (70).

### 2.11 Histopathological analysis

At the end of the experiment, the collected intestinal tissues were immediately fixed in 10% buffered neutral formalin solution for 24 h, sectioned, rinsed in freshly distilled water, dehydrated in graded ascending ethanol (70%, 80%, 90%, 95%, and 100%), clarified in xylene, and ultimately embedded in paraffin wax. The longitudinal and transverse thin sections (5 mm thick) of paraffin-embedded tissues were sliced using a Rotatory Microtom microtome (Leica RM 2155, England), then stained with hematoxylin and eosin (H&E) (71), and subsequently investigated under a light microscope equipped with a digital camera (72). Stained slides were examined, and the tissue lesions were subsequently recorded.

Furthermore, the intestinal histomorphometry, including intestinal villi length (VL), villi width (VW), absorption surface area (ASA), and intestinal crypt depth (CD), was estimated. These metrics were assessed for each individual over 50 well-aligned villi and corresponding crypts from each section of all intestinal segments and averaged for each broiler. The heights of the villi were measured from their tip to the base, and the widths were assessed at the midpoint of their height. The tissue sections were analyzed using a light microscope fitted with a full HD microscopic camera and image analysis software (Leica Microsystem, Germany). The metrics were measured by image analysis software for statistical analysis. The ASA was computed as follows: ASA (mm^2^) = villus height × villus width (73).

### 2.12 Statistical analysis

The general linear model approach of the SPSS Inc. software version 20 (IBM Corp., NY, USA) was utilized to analyze all of the data after Levene's test confirmed the homogeneity between treatment and Shapiro-Wilk's test confirmed normality. Statistical significance (*p* < 0.05) among the experimental groups was assessed using One-way ANOVA and Tukey's *post-hoc* tests. The graphics used in the present research were created via the GraphPad Prism program v.8 (San Diego, USA).

## 3 Results

### 3.1 Growth performance and mortality rates

Table 3 depicts the impact of dietary incorporation of *Salmonella* BP, GSOPs, and their combination on the broilers' growth performance. Throughout the starter phase, the metrics of body weight gain, and feed conversion ratio were significantly enhanced (*p* < 0.01) in groups receiving dietary GSOPs (397.76 g/bird and 1.256, respectively), and GSOPs+BP (397.72 g/bird and 1.246, respectively) inclusion, in contrast to the control groups (380.08 g/bird and 1.276, respectively) with no significant difference between them. Over the course of the grower phase, the BWG and FCR were markedly enhanced (*p* < 0.01) in broilers receiving dietary BP, GSOPs, and GSOPs+BP supplementations, when compared with broilers in the infective control group. Broilers in the GSOPs+BP group revealed the most substantial increase (*p* < 0.01) in the BWG (1,053.474 g/bird) through the grower period in comparison to broilers on the control diet (730.82 g/bird), even following *S*. Typhimurium challenge. The FCR was substantially decreased (*p* < 0.01) in GSOPs+BP (1.886d) than in BP, and GSOPs-supplemented groups (2.214 and 1.99, respectively) during the finisher period in comparison to the IC group (2.426), which suggests the synergistic impact between BP and GSOPs. During the finisher phase, broilers in GSOPs and BP groups showed a significant (*p* < 0.01) elevation in the BWG, unlike the IC group. Throughout the entire growing period of 36 days, the impaired BWG, FCR, and elevated mortality percentages observed in groups challenged with *S*. Typhimurium were significantly improved in those receiving a combination of GSOPs and BP, followed by the GSOPs and BP groups, which suggests the synergistic impact between BP and GSOPs. Through the overall rearing period, broilers offered the combination of dietary GSOPs and BP revealed substantial improvement in BWG (2,293 g/bird), and FCR (1.696), with the lowest mortality rate (4%), unlike the IC group (1,877.4 g/bird, 2.06, and 40%, respectively).

**Table 3:** Impact of dietary *Salmonella* bacteriophage (BP), grape seed oligomeric pro-anthocyanidins (GSOPs), and their combinations on growth performance attributes in challenged broilers.

**Parameter**	**Experimental group**					**SEM**	***p-*value**
	**C**	**IC**	**BP**	**GSOPs**	**BP**+**GSOPs**		
**Starter (1–11 d)**							
Initial BW	48.4	48.2	47.8	48.8	48.6	0.17	0.99
BW, g/bird	354.48^b^	355.14^b^	352.52^b^	364.78^a^	367.7^a^	3.11	< 0.001
BWG, g/bird	380.08^c^	388.4^b^	389.16^b^	397.76^a^	397.72^a^	1.2	< 0.001
FI, g/bird	297.12^c^	306.74^b^	304.72^b^	315.98^a^	319.1^a^	3.2	0.001
FCR	1.279^a^	1.266^ab^	1.277^a^	1.256^bc^	1.246^c^	< 0.001	< 0.001
**Grower (12–21 d)**							
BW, g/bird	1,449.96^a^	1,085.8^c^	1,282.33^b^	1,315.4^b^	1,421.2^a^	23.1	< 0.001
BWG, g/bird	1,095.48^a^	730.82^c^	929.81^b^	950.54^b^	1,053.474^a^	60.6	< 0.001
FI, g/bird	1,675.4^b^	1,446.4^c^	1,595.66^b^	1,630.4^b^	1,769.6^a^	23.2	< 0.001
FCR	1.529^c^	1.984^a^	1.716^b^	1.716^b^	1.682^b^	< 0.001	< 0.001
**Finisher (22–35 d)**							
BW, g/bird	2,350.72^a^	1,925.8^c^	2,260.39^b^	2,281.4^b^	2,293^ab^	22.6	< 0.001
BWG, g/bird	900.76^bc^	839.9^c^	978.07^a^	966.36^ab^	871.76^c^	65	< 0.001
FI, g/bird	1,773.6^cd^	2,037.4^ab^	2,165.48^a^	1,924.4^bc^	1,643.8^d^	21.6	< 0.001
FCR	1.969^c^	2.426^a^	2.214^b^	1.99^c^	1.886^d^	< 0.001	< 0.001
**Allover**							
BW, g/bird	2,350.72^a^	1,925.8^c^	2,260.39^b^	2,281.4^b^	2,293^ab^	26.5	< 0.001
BWG, g/bird	2,293.36^a^	1,877.4^c^	2,212.59^b^	2,232.6^ab^	2,244.4^ab^	6.5	< 0.001
FI, g/bird	3,829.1^b^	3,871.8^b^	4,150.29^a^	3,951.6^ab^	3,811.2^b^	27.3	< 0.001
FCR	1.669^d^	2.06^a^	1.876^b^	1.77^c^	1.696^d^	< 0.001	< 0.001
Cumulative mortality, %	0^c^	40^a^	12^b^	6^bc^	4^bc^	< 0.001	< 0.001

BW, body weight; BWG, body weight gain; FI, feed intake; FCR, feed conversion ratio.

Results are displayed as mean ± SEM (standard error of the mean).

^a − d^Mean values denoted by various letters in the same row exhibit significant differences at p < 0.05. C (negative control): chicks were administered a basal diet devoid of any additives and were not subjected to any challenge, IC (infective control): chicks were offered a basal diet without supplements and were challenged with S. Typhimurium, BP: chicks were offered a basal diet augmented with Salmonella bacteriophage (BP) alone at concentrations of 10^9^ PFU/0.1 ml, GSOPs: chicks were offered a basal diet augmented with grape seed oligomeric pro-anthocyanidins (GSOPs) alone at a level of 400 mg/kg diet, and GSOPs+BP: chicks were offered a basal diet augmented with combination of both Salmonella BP and GSOPs. The IC and three treatment groups were challenged at 14 days of age (grower phase) with MDR S. Typhimurium strain.

### 3.2 Antioxidant potential of *S*. Typhimurium challenged broilers in response to dietary bacteriophage, and grape seed oligomeric pro-anthocyanidins

Data regarding the influence of dietary fortification with GSOPs, *Salmonella* BP, and their combination on the intestinal oxidative and antioxidant attributes in broilers challenged with *S*. Typhimurium are emphasized in Table 4. The incorporation of GSOPs, *Salmonella* BP, and their combination in broiler diets significantly reduced (*p* < 0.01) the levels of oxidative stress markers (H_2_O_2_, ROS, and MDA), and elevated the antioxidant enzyme activity (CAT, SOD, and GSH-Px) compared to the IC group, at 7 dpc with *S*. Typhimurium. Dietary fortification with BP, GSOPs, and their combination mitigated the negative impact of *S*. Typhimurium infection on the oxidative and antioxidant attributes and restored their activities to levels comparable to those in the negative control group. Furthermore, the activity of CAT, SOD, and GSH-Px antioxidant enzymes (136.48, 122.37, and 166.82 U/mg, respectively) reached their maxima (*p* < 0.01) after dietary fortification with a combination of BP and GSOPs when compared with the IC group (96.2, 49.63, and 120.35 U/mg, respectively), at 7 dpc. Simultaneously, the lowest levels of H_2_O_2_, ROS, and MDA oxidative stress biomarkers were notably recorded (*p* < 0.05) in the BP+GSOPs group (1.47 μmol/g, 22.65 μl/g, and 1.47 nmol/ml, respectively), unlike the IC group (6.99 μmol/g, 63.88 μl/g, and 21.11 nmol/ml, respectively), at 7 dpc.

**Table 4:** Impact of dietary *Salmonella* bacteriophage (BP), grape seed oligomeric pro-anthocyanidins (GSOPs), and their combinations on the level of intestinal oxidative and antioxidant markers post-challenge with *S*. Typhimurium.

**Parameters**	**Experimental group**					***p*-value**	**SEM**
	**C**	**IC**	**BP**	**GSOPs**	**BP**+**GSOPs**		
ROS (μl/g tissue)	30.21^b^	63.88^a^	29.48^b^	25.38^c^	22.65^c^	< 0.001	9.12
H_2_O_2_ (μmol/g tissue)	2.42^b^	6.99^a^	2.13^c^	1.61^d^	1.47 ^d^	< 0.001	0.11
MDA (nmol/ml)	8.84^b^	21.11^a^	7.99^bc^	7.21^bc^	6.13^c^	< 0.001	1.4
SOD (U/mg)	49.63^d^	77.99^c^	87.39^b^	121.16^a^	122.37^a^	< 0.001	11.6
GSH-Px (U/mg)	120.35^c^	140.28^b^	134.99^b^	160.82^a^	166.82^a^	< 0.001	12.3
CAT (U/mg)	96.2^c^	95.81^c^	118.72^b^	132.15^a^	136.48^a^	< 0.001	10.2

ROS, reactive oxygen species; H_2_O_2_, hydrogen peroxide; MDA, malondialdehyde; SOD, superoxide dismutase; GSH-Px, glutathione peroxidase; CAT, catalase.

Results are displayed as means ± SEM (standard error of the mean).

^a − d^Mean values denoted by various letters in the same row exhibit significant differences at *p* < 0.05. **C** (negative control): chicks were administered a basal diet devoid of any additives and were not subjected to any challenge, IC (infective control): chicks were offered a basal diet without supplements and were challenged with S. Typhimurium, BP: chicks were offered a basal diet augmented with Salmonella bacteriophage (BP) alone at concentrations of 10^9^ PFU/0.1 ml, GSOPs: chicks were offered a basal diet augmented with grape seed oligomeric pro-anthocyanidins (GSOPs) alone at a level of 400 mg/kg diet, and GSOPs+BP: chicks were offered a basal diet augmented with combination of both Salmonella BP and GSOPs. The IC and three treatment groups were challenged at 14 days of age with MDR S. Typhimurium strain.

### 3.3 Analysis of serum biochemical and immunological-related parameters

Table 5 illustrates the findings of the serum biochemical and immunological markers following dietary supplementation with GSOPs, *Salmonella* BP, and their combination in broilers challenged with *S*. Typhimurium. At 7 days following the *S*. Typhimurium challenge, the unsupplemented and challenged broilers demonstrated significant (*p* < 0.05) decreased levels of creatinine, urea, AST, and ALT, in comparison with the control negative group. Concurrently, dietary BP and GSOPs fortification significantly (*p* < 0.05) restored ALT (26.85 and 23.37 U/L, respectively) and creatinine (0.503 and 0.407 mg/dl, respectively) concentrations in comparison to the IC group (18.5 U/L and 0.283 mg/dl, respectively). Broilers offered bacteriophage inclusion significantly enhanced urea concentration (6.12 mg/dl) regarding the infective control group (3.57 mg/dl). There was no significant difference in the activity of AST among the three treatment groups and the control negative group.

**Table 5:** Efficacy of dietary *Salmonella* bacteriophage (BP), grape seed oligomeric pro-anthocyanidins (GSOPs), and their combinations on the levels of serum biochemical and immune-related parameters in broilers post-challenge with *S*. Typhimurium.

**Parameters**	**Experimental group**					***p*-value**	**SEM**
	**C**	**IC**	**BP**	**GSOPs**	**BP**+**GSOPs**		
Creatinine (mg/dl)	0.587^a^	0.283^c^	0.503^a^	0.407^b^	0.363^bc^	< 0.001	0.11
Urea (mg/dl)	8.65^a^	3.57^c^	6.12 ^b^	4.57^c^	3.54^c^	< 0.001	0.13
AST (U/L)	60.24^a^	31.5^bc^	34.71^b^	32.63^b^	27.34^c^	< 0.001	0.48
ALT (U/L)	28.93^a^	18.5^d^	26.85^ab^	23.37^bc^	21.1^cd^	< 0.001	0.6
LYZ (U/ml)	160.21^d^	212.1^a^	199.59^b^	192.05^b^	176.95^c^	< 0.001	20.03
NO (μmol/L)	2.8^d^	6.83^a^	6.01^b^	5.77^b^	5.27^c^	< 0.001	0.02
CRP (mg/L)	1.17^d^	3.87^a^	2.84 ^b^	2.43^b^	1.69^c^	< 0.001	0.21
Complement C3	1.13^d^	2.35^a^	1.97^b^	1.77^b^	1.51^c^	< 0.001	0.22
IgG (mg/dl)	13^c^	11.87^d^	14.53^b^	14.82^b^	15.53^a^	< 0.001	0.14
IgM (mg/dl)	16.15^b^	15.53^b^	17.49^a^	17.82^a^	18.52^a^	< 0.001	0.23

AST, aspartate transaminase; ALT, alanine transaminase; LYZ, lysozyme; NO, nitric oxide; Ig, immunoglobulin; CRP, c-reactive protein.

Results are displayed as means ± SEM (standard error of the mean).

^a − d^Mean values denoted by various letters in the same row exhibit significant differences at *p* < 0.05. **C** (negative control): chicks were administered a basal diet devoid of any additives and were not subjected to any challenge, IC (infective control): chicks were offered a basal diet without supplements and were challenged with S. Typhimurium, BP: chicks were offered a basal diet augmented with Salmonella bacteriophage (BP) alone at concentrations of 10^9^ PFU/0.1 ml, GSOPs: chicks were offered a basal diet augmented with grape seed oligomeric pro-anthocyanidins (GSOPs) alone at a level of 400 mg/kg diet, and GSOPs+BP: chicks were offered a basal diet augmented with combination of both Salmonella BP and GSOPs. The IC and three treatment groups were challenged at 14 days of age with MDR S. Typhimurium strain.

In the first week after the *S*. Typhimurium challenge, the administration of dietary BP, GSOPs, and their combination markedly (*p* < 0.01) improved the immunological reactions of birds following challenge with *S*. Typhimurium by diminished the activities of serum LYZ, NO, CRP, and complement C3 levels, while augmenting serum IgG, and IgM levels, in comparison to the IC group. At 7 dpc, birds offered dietary BP, and GSOPs combination exhibited the most substantial (*p* < 0.01) immunological reaction, as indicated by reduction in the serum concentrations of LYZ, NO, CRP, and complement C3 (176.95 U/ml, 5.27 μmol/L, 1.69 mg/L, and 1.51, respectively), alongside increased serum IgG level (15.53 mg/dl), unlike the IC group (212.1 U/ml, 6.83 μmol/L, 3.87 mg/L, 2.35, and 11.87 mg/dl, respectively), which suggest synergistic positive impact between BP, and GSOPs. Additionally, at 7 dpc, the BP+GSOPs group exhibited the highest IgM value (18.52 mg/dl) in comparison to the IC group (15.53 mg/dl), with no significant differences observed between the BP, GSOPs, and BP+GSOPs groups.

### 3.4 Quantification of cecal microbial populations by quantitative real-time PCR assay

**Figure 1:**
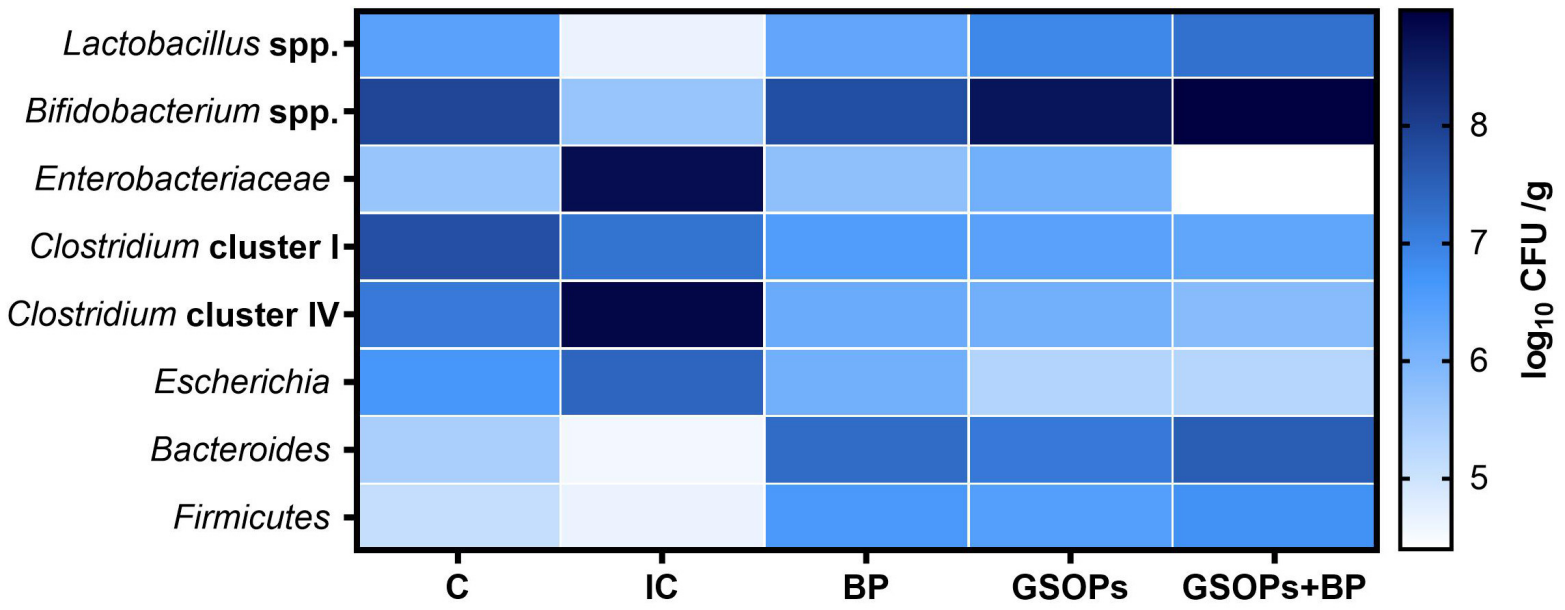
Efficacy of dietary *Salmonella* bacteriophage (BP), grape seed oligomeric pro-anthocyanidins (GSOPs), and their combinations on the quantity of cecal *Lactobacillus* spp., *Bifidobacterium* spp., *Bacteroides, Firmicutes, Escherichia, Enterobacteriaceae, Clostridium* clusters I, and IV in the cecal content of broilers at 7 days post-challenge with *S*. Typhimurium strain as determined by qPCR technique. **C** (negative control): chicks were administered a basal diet devoid of any additives and were not subjected to any challenge, IC (infective control): chicks were offered a basal diet without supplements and were challenged with *S*. Typhimurium, BP: *S*. Typhimurium challenged chicks were offered a basal diet augmented with *Salmonella* bacteriophage (BP) alone at concentrations of 10^9^ PFU/0.1 ml, GSOPs: *S*. Typhimurium challenged chicks were offered a basal diet augmented with grape seed oligomeric pro-anthocyanidins (GSOPs) alone at a level of 400 mg/kg diet, and GSOPs+BP: *S*. Typhimurium challenged chicks were offered a basal diet augmented with combination of both *Salmonella* BP and GSOPs.

Figure 1 displays the quantification outcomes of cecal bacterial populations at 7 dpc with S. Typhimurium using the qPCR assay. At 7 dpc with S. Typhimurium, the pathogenic bacteria, including Enterobacteriaceae, Escherichia, Clostridium clusters I and IV, were numerically and substantially (p < 0.05) reduced in the cecal contents of birds fortified with dietary Salmonella BP, GSOPs, and their combinations, regarding the IC group. Meanwhile, the incorporation of dietary Salmonella BP, GSOPs, and their combinations led to a significant (p < 0.05) increase in the abundance of cecal beneficial bacteria, including Bacteroides, Firmicutes, Bifidobacterium, and Lactobacillus spp., unlike the IC group, at 7 dpc with S. Typhimurium. Post-supplementation with a combination of BP and GSOPs, the cecal contents of birds exhibited the highest populations of Lactobacillus spp. (7.2 log_10_ CFU/g), alongside the lowest populations of Enterobacteriaceae (4.4 log_10_ CFU/g), and Clostridium clusters IV (5.87 log_10_ CFU/g), unlike the infective control group (4.65, 8.76 and 8.87 log_10_ CFU/g), at 7 dpc, which indicates a potential synergistic impact between BP and GSOPs. At 7 dpc, the most substantial (p < 0.05) increase in the populations of Bacteroides (7.32 and 7.54 log_10_ CFU/g), and Firmicutes (6.61 and 6.75 log_10_ CFU/g) were determined in the cecal contents of broilers offered dietary BP, and BP+GSOPs, respectively, unlike the IC group (4.54 and 4.65 log_10_ CFU/g, respectively), with no significant differences between the two treatments. Furthermore, broilers in GSOPs, and GSOPs+BP groups showed the highest Bifidobacterium cecal populations (8.65 and 8.98 log_10_ CFU/g, respectively), and the lowest populations of Escherichia (5.32 and 5.29 log_10_ CFU/g, respectively), and Clostridium clusters IV (5.87 log_10_ CFU/g), unlike the IC group (5.65 and 7.43 log_10_ CFU/g, respectively), at 7 dpc, with no significant differences between them.

### 3.5 Quantification of cecal *Salmonella* Typhimurium DNA copies

**Figure 2:**
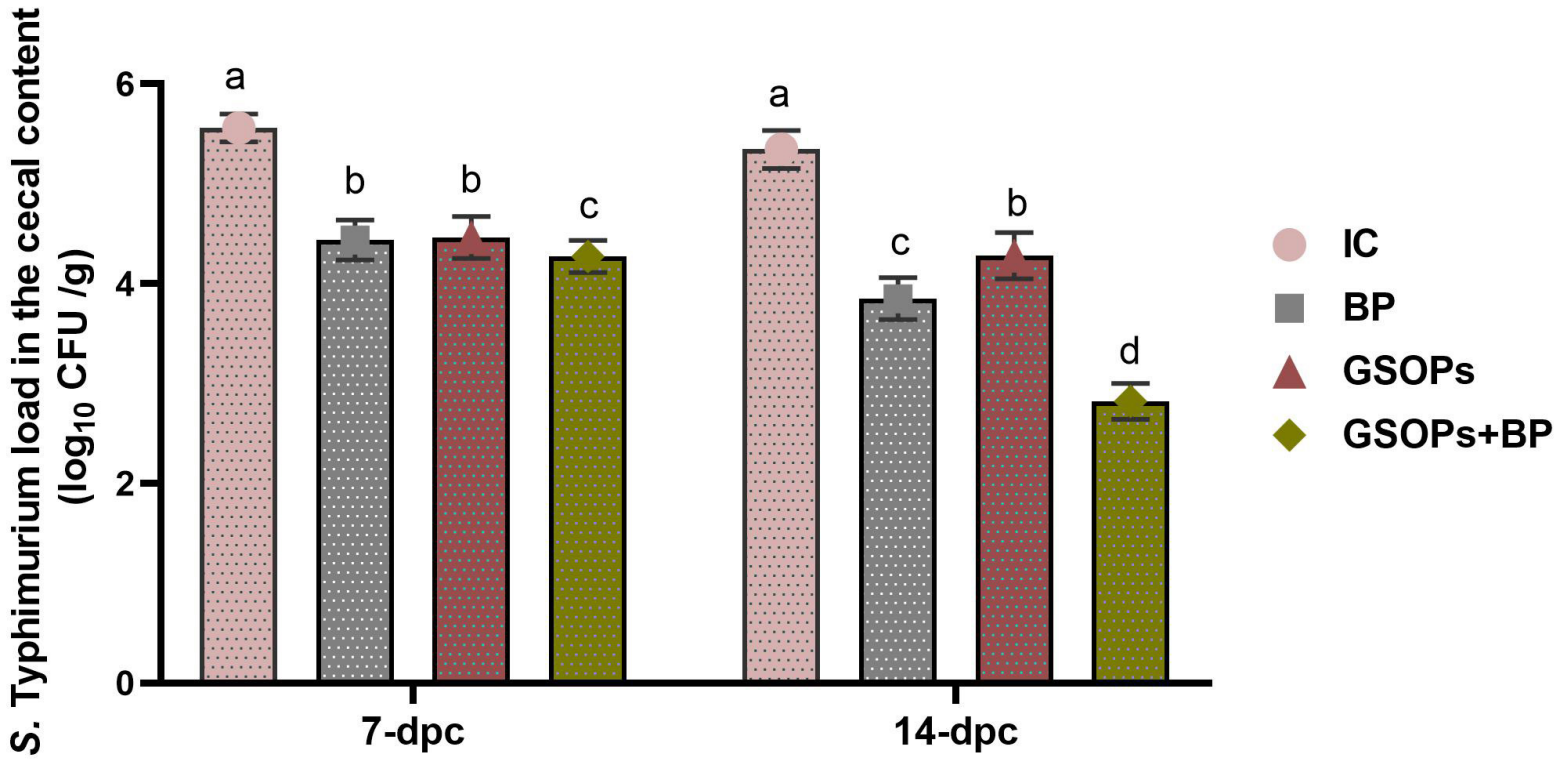
Quantification of *Salmonella* Typhimurium populations in the cecal content of broilers following dietary *Salmonella* bacteriophage (BP), grape seed oligomeric pro-anthocyanidins (GSOPs), and their combinations at 7 and 14 days post-challenge (dpc) with *S*. Typhimurium strain using qPCR assay. Results are presented as means ± standard error of the mean (SEM). ^a − d^ Values within the same column with differing superscripts are substantially different at *p* < 0.05. IC (infective control): chicks were offered a basal diet without supplements and were challenged with *S*. Typhimurium, BP: *S*. Typhimurium challenged chicks were offered a basal diet augmented with *Salmonella* bacteriophage (BP) alone at concentrations of 10^9^ PFU/0.1 ml, GSOPs: *S*. Typhimurium challenged chicks were offered a basal diet augmented with grape seed oligomeric pro-anthocyanidins (GSOPs) alone at a level of 400 mg/kg diet, and GSOPs+BP: *S*. Typhimurium challenged chicks were offered a basal diet augmented with combination of both *Salmonella* BP and GSOPs.

Figure 2 illustrates the quantification results of *S*. Typhimurium in the cecal contents of challenged broilers at 7 and 14-pdc with the MDR *S*. Typhimurium strain. The quantitative investigation of cecal *S*. Typhimurium post-challenge revealed that all treated groups had significantly fewer *Salmonella* populations compared to the infective control group, with a consistent decline in counts over time. The incorporation of dietary BP, GSOPs, and their combination considerably (*p* < 0.05) diminished *S*. Typhimurium abundance at 7 and 14-dpc in comparison to the IC group. Significantly, our results indicated that *S*. Typhimurium abundance were at their lowest levels in the cecal contents of challenged birds offered a combination of BP, and GSOPs at 7, and 14-dpc with *S*. Typhimurium strain (4.27, and 2.82 log_10_ CFU/g, respectively) compared to the IC group (5.56, and 5.34 log_10_ CFU/g, respectively), which indicate a possible synergistic impact between BP, and GSOPs.

### 3.6 Expression profiles of *Salmonella* Typhimurium virulence genes

**Figure 3:**
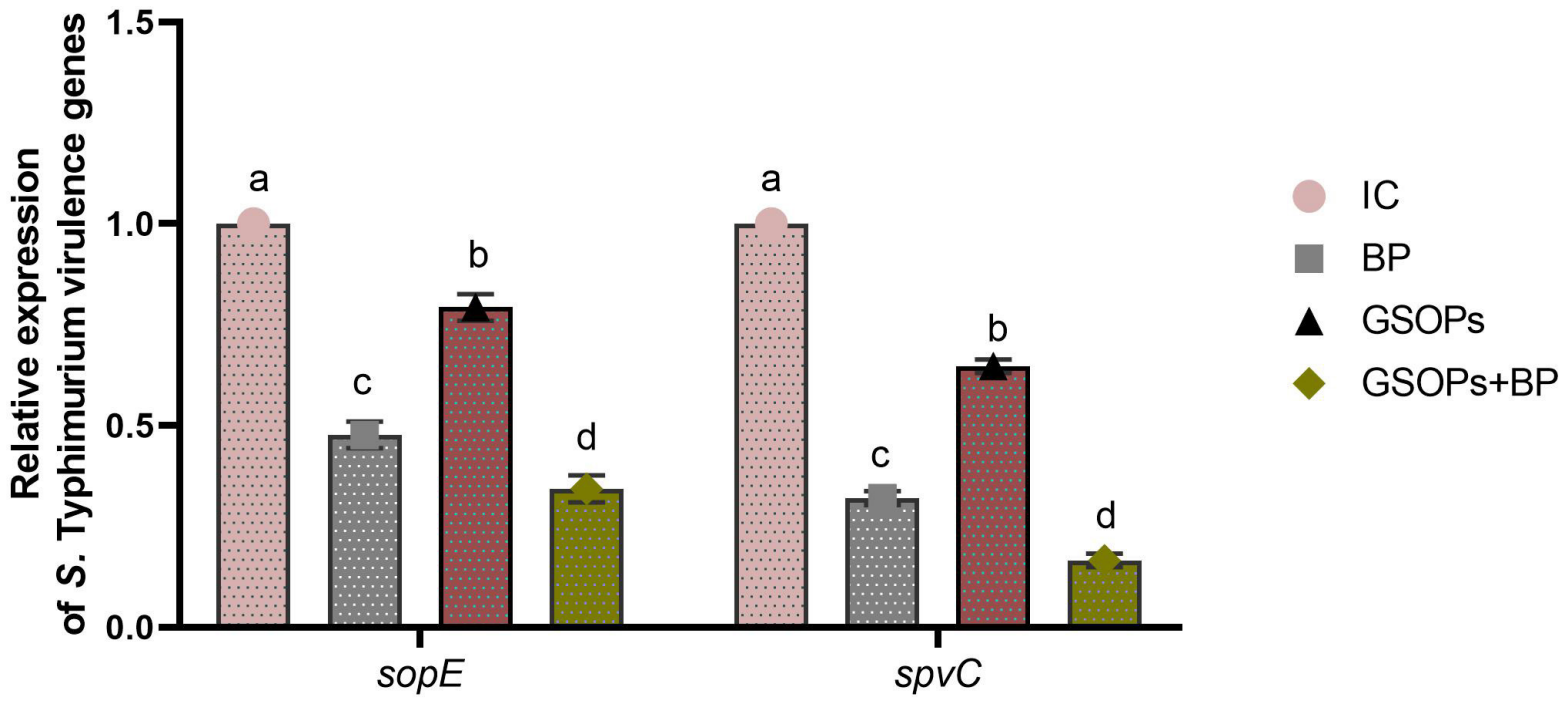
Gene expression levels of *Salmonella Typhimurium* virulence-related genes [*sopE* (*Salmonella* outer protein E), and *spvC* (*Salmonella* plasmid virulence C)] in the cecal tissues of challenged broiler chickens administered dietary *Salmonella* bacteriophage (BP), grape seed oligomeric pro-anthocyanidins (GSOPs), and their combinations at 7 days post-challenge with *S*. Typhimurium strain, as determined by RT-qPCR assay. Results are presented as means ± standard error of the mean (SEM). ^a − d^ Values within the same column with differing superscripts are substantially different at *p* < 0.05. IC (infective control): chicks were offered a basal diet without supplements and were challenged with *S*. Typhimurium, BP: *S*. Typhimurium challenged chicks were offered a basal diet augmented with *Salmonella* bacteriophage (BP) alone at concentrations of 10^9^ PFU/0.1 ml, GSOPs: *S*. Typhimurium challenged chicks were offered a basal diet augmented with grape seed oligomeric pro-anthocyanidins (GSOPs) alone at a level of 400 mg/kg diet, and GSOPs+BP: *S*. Typhimurium challenged chicks were offered a basal diet augmented with combination of both *Salmonella* BP and GSOPs.

Figure 3 reveals the impact of dietary BP, GSOPs, and their combinations on the expression of *S*. Typhimurium *sopE* and *spvC* virulence genes in the cecal tissues of challenged birds, as assessed by RT-qPCR at 7 dpc with the *S*. Typhimurium strain. The results indicated that BP, GSOPs, and their combinations considerably (*p* < 0.05) reduced the transcript levels of *S*. Typhimurium *sopE* and *spvC* virulence genes, unlike the IC group at 7 dpc with S. Typhimurium. When compared with the IC group, the most pronounced (*p* < 0.05) downregulation in *S*. Typhimurium *sopE*, and *spvC* transcript levels was detected in the cecal tissues of GSOPs+BP (0.34 and 0.17-fold change, respectively), followed by BP (0.47 and 0.32-fold change, respectively), then GSOPs (0.79 and 0.65-fold change, respectively) fed birds, at 7 dpc, which indicates a potential synergistic impact between BP and GSOPs.

### 3.7 Expression analysis of genes related to intestinal barriers post-infection with *Salmonella* Typhimurium

**Figure 4:**
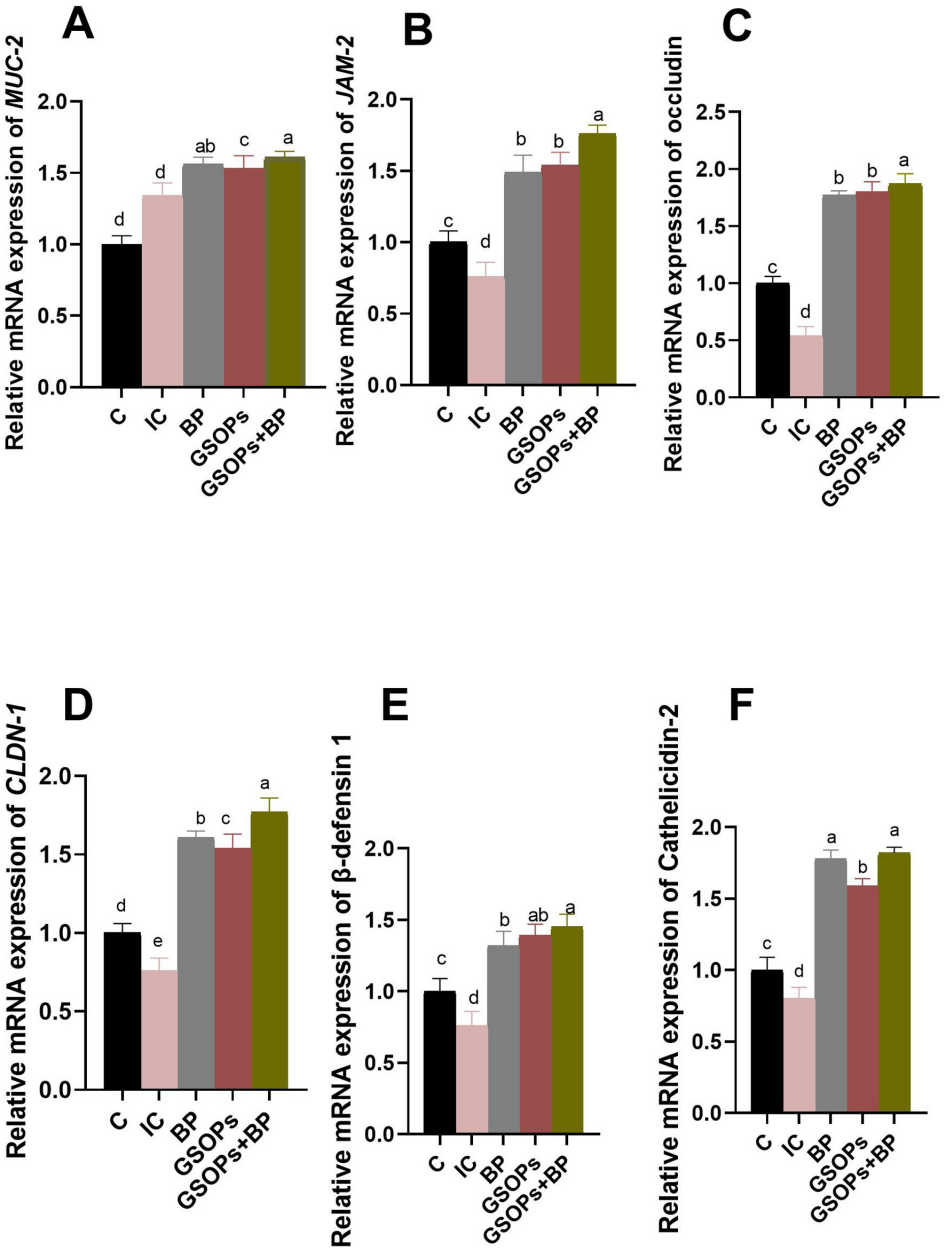
RT-qPCR analysis of the relative transcript levels of genes related to barrier functions [*MUC*-2 (mucin-2, **A**), *JAM*-2 (junctional adhesion molecule-2, **B**), occludin **(C)**, *CLDN*-1 (claudins-1, **D**), β-defensin-1 **(E)**, and cathelicidins-2 **(F)**] in the cecal tissues of challenged broilers fortified with dietary *Salmonella* bacteriophage (BP), grape seed oligomeric pro-anthocyanidins (GSOPs) and their combinations at 7 days post-challenge with *S*. Typhimurium strain, as determined by RT-qPCR assay. Results are presented as means ± standard error of the mean (SEM). ^a − d^ Values within the same column with differing superscripts are substantially different at *p* < 0.05. **(C)** (negative control): chicks were administered a basal diet devoid of any additives and were not subjected to any challenge, IC (infective control): chicks were offered a basal diet without supplements and were challenged with *S*. Typhimurium, BP: *S*. Typhimurium challenged chicks were offered a basal diet augmented with *Salmonella* bacteriophage (BP) alone at concentrations of 10^9^ PFU/0.1 ml, GSOPs: *S*. Typhimurium challenged chicks were offered a basal diet augmented with grape seed oligomeric pro-anthocyanidins (GSOPs) alone at a level of 400 mg/kg diet, and GSOPs+BP: *S*. Typhimurium challenged chicks were offered a basal diet augmented with combination of both *Salmonella* BP and GSOPs.

Figure 4 illustrates the expression profiles of genes encoding intestinal barriers in the cecal tissues of challenged broilers offered dietary BP, GSOPs, and their combinations at 7 dpc with *S*. Typhimurium strain. At 7 dpc with *S*. Typhimurium, dietary fortification with BP, GSOPs, and their combination considerably (*p* < 0.05) elevated the transcript levels of genes related to tight junction proteins (TJPs) [*JAM-2*, CLDN-1, and occludin], and mucins [*MUC-2*], host defense peptides (HDP) [cathelicidins-2, and β-defensin-1], with respect to the infective control group. The most substantial (*p* < 0.05) elevation in the expression levels of genes related to TJP, including CLDN-1, *JAM-2*, and occludin genes were recorded in the group receiving BP, and GSOPs combination (1.76, 1.54, and 1.87-fold change, respectively), unlike the IC group at 7 dpc, which indicate a potential synergistic impact between BP and GSOPs. Furthermore, the most substantial (*p* < 0.05) upregulations in the expression of the *MUC-2* and cathelicidins-2 genes were detected in BP (1.56 and 1.78-fold change, respectively), and GSOPs+BP (1.61 and 1.82-fold change, respectively), with no significant difference between them, compared with the IC group at 7 dpc. The most significant (*p* < 0.05) overexpression of the β-defensin-1 gene was observed in broilers administered GSOPs and GSOPs+BP (1.39- and 1.45-fold change, respectively) regarding the IC group at 7 dpc. The *S*. Typhimurium challenge did not adversely affect the relative transcript levels of genes associated with barrier functions following dietary inclusion with BP, GSOPs, and their combinations, as indicated by their significantly higher levels (*p* < 0.05), particularly in the GSOPs+BP group.

### 3.8 Regulation of genes related to autophagy post-challenge with *Salmonella* Typhimurium

**Figure 5:**
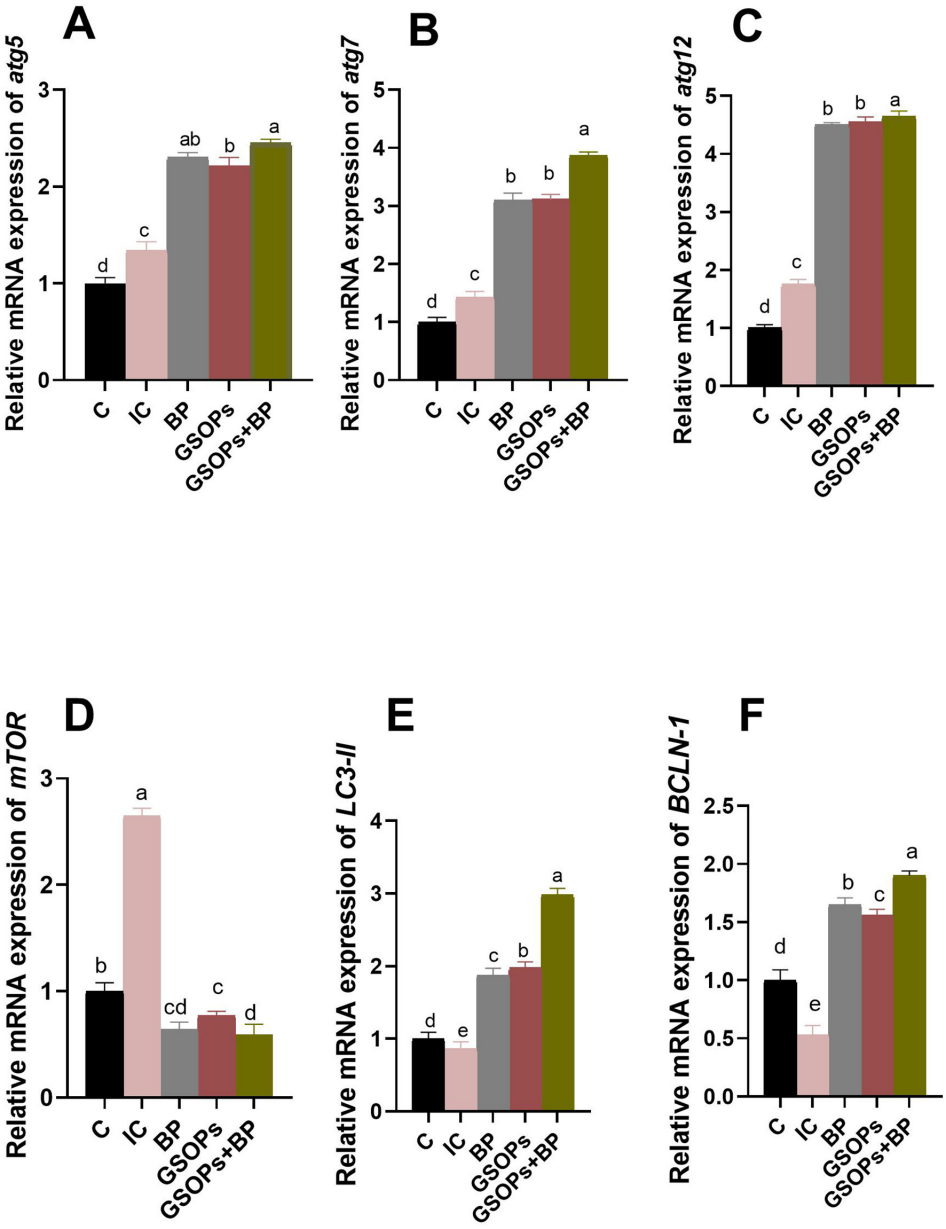
Transcriptional levels of autophagy-related genes [autophagy (*atg5*, **A**), *atg7* **(B)**, *atg12* **(C)**, mechanistic target of rapamycin (*mTOR*, **D**), microtubule-associated proteins 1A/1B light chain (*LC3-II*, **E**), and beclin-1 (*BCLN-1*, **F**)] in the cecal tissues of challenged broilers supplemented with dietary *Salmonella* bacteriophage (BP), grape seed oligomeric pro-anthocyanidins (GSOPs) and their combinations at 7 days post-challenge with *S*. Typhimurium strain, as determined by RT-qPCR assay. Results are presented as means ± standard error of the mean (SEM). ^a − d^ Values within the same column with differing superscripts are substantially different at *p* < 0.05. C (negative control): chicks were administered a basal diet devoid of any additives and were not subjected to any challenge, IC (infective control): chicks were offered a basal diet without supplements and were challenged with *S*. Typhimurium, BP: *S*. Typhimurium challenged chicks were offered a basal diet augmented with *Salmonella* bacteriophage (BP) alone at concentrations of 10^9^ PFU/0.1 ml, GSOPs: *S*. Typhimurium challenged chicks were offered a basal diet augmented with grape seed oligomeric pro-anthocyanidins (GSOPs) alone at a level of 400 mg/kg diet, and GSOPs+BP: *S*. Typhimurium challenged chicks were offered a basal diet augmented with combination of both *Salmonella* BP and GSOPs.

The effectiveness of incorporating dietary BP, GSOPs, and their combination on the transcript levels of autophagy-related genes following *S*. Typhimurium challenge is depicted in Figure 5. In comparison to the IC group, the inclusion of BP, GSOPs, and their combination in broilers' diet significantly (*p* < 0.05) downregulated *mTOR* expression, alongside upregulating the transcriptional levels of *BCLN-1, LC3-II, atg5, atg7*, and *atg12* genes in the cecal tissues of challenged broilers at 7 dpc with *S*. Typhimurium strain. Significantly, birds administered a combination of BP, and GSOPs exhibited the greatest (*p* < 0.05) transcript levels of cecal *atg7, atg12, LC3-II*, and *BCLN-1* (3.87, 4.65, 2.98, and 1.9-fold change, respectively) genes, and the lowest (*p* < 0.05) *mTOR* gene (0.59-fold change) expression relative to the infective control group at 7 dpc, which indicate a possible synergistic impact between BP, and GSOPs. Furthermore, the most considerable (*p* < 0.05) elevation in the *atg5* transcriptional level was observed among birds received dietary GSOPs, and BP combination (2.45-fold change), followed by BP group (2.3-fold change), with no significant differences between them, unlike the control negative group at 7 dpc.

### 3.9 Regulation of immune response-related genes post-challenge with *Salmonella* Typhimurium

**Figure 6:**
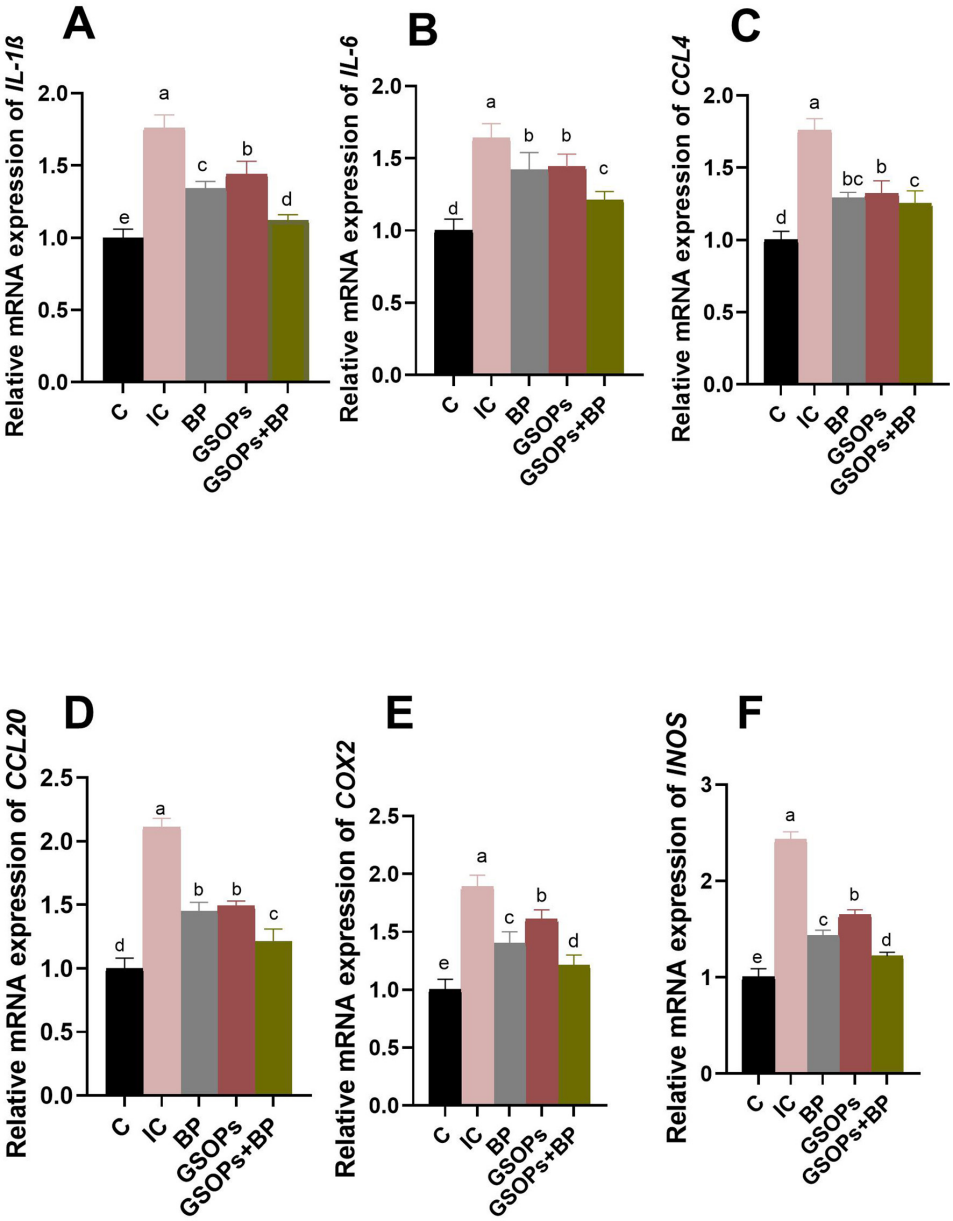
The effectiveness of dietary inclusion with *Salmonella* bacteriophage (BP), grape seed oligomeric pro-anthocyanidins (GSOPs) either individually or in combination on the expression levels of interleukin-6 (*IL-1*β, **A**), *IL-6* **(B)**, chemokine C–C motif ligand 4 (*CCL4*, **C**), *CCL20* **(D)**, cyclooxygenase-2 (*COX-2*, **E**), and inducible nitric oxide synthase (*iNOS*, **F**) genes in the intestinal tissues of broilers at 7-days post-challenge with *S*. Typhimurium strain as determined by RT-qPCR assay. Results are presented as means ± standard error of the mean (SEM). ^a − d^ Values within the same column with differing superscripts are substantially different at *p* < 0.05. C (negative control): chicks were administered a basal diet devoid of any additives and were not subjected to any challenge, IC (infective control): chicks were offered a basal diet without supplements and were challenged with *S*. Typhimurium, BP: *S*. Typhimurium challenged chicks were offered a basal diet augmented with *Salmonella* bacteriophage (BP) alone at concentrations of 10^9^ PFU/0.1 ml, GSOPs: *S*. Typhimurium challenged chicks were offered a basal diet augmented with grape seed oligomeric pro-anthocyanidins (GSOPs) alone at a level of 400 mg/kg diet, and GSOPs+BP: *S*. Typhimurium challenged chicks were offered a basal diet augmented with combination of both *Salmonella* BP and GSOPs.

The findings illustrated in Figure 6 demonstrated differential transcriptional levels of intestinal immune response and inflammation-related markers at 7 dpc with *S*. Typhimurium, in response to the incorporation of dietary BP, GSOPs, or their combination. At 7 dpc with *S*. Typhimurium, dietary fortification with BP, GSOPs, or their combination significantly (*p* < 0.05) minimized the heightened inflammatory response subsequent to *S. Typhimurium* challenge. At 7 dpc with S. Typhimurium, incorporation of dietary BP, GSOPs, or their combination significantly (*p* < 0.05) downregulated the mRNA expression levels of genes encoding proinflammatory cytokines (*IL-6* and *IL-1*β), chemokines (*CCL4* and *CCL20*), alongside *COX-2* and *iNOS* genes, in the IC group. The most significant downregulation of *IL-1*β*, IL-6, CCL20, COX-2*, and *iNOS* genes was recorded in the groups administered a combination of BP, and GSOPs (1.12, 1.21, 1.21, 1.21, and 1.22-fold change, respectively), unlike the IC group (1.76, 1.44, 2.11, 1.89, and 2.43-fold change, respectively), which indicate a potential synergistic effect between BP and GSOPs. Furthermore, the transcriptional level of the *CCL4* gene was considerably downregulated (*p* < 0.05) in broilers administered a combination of BP and GSOPs (1.25-fold change), followed by those receiving BP alone (1.29-fold change), in contrast to the infected control group (1.766-fold change), with no significant difference between the two groups.

### 3.10 Histopathological alteration post-challenge with *Salmonella* Typhimurium

**Figure 7:**
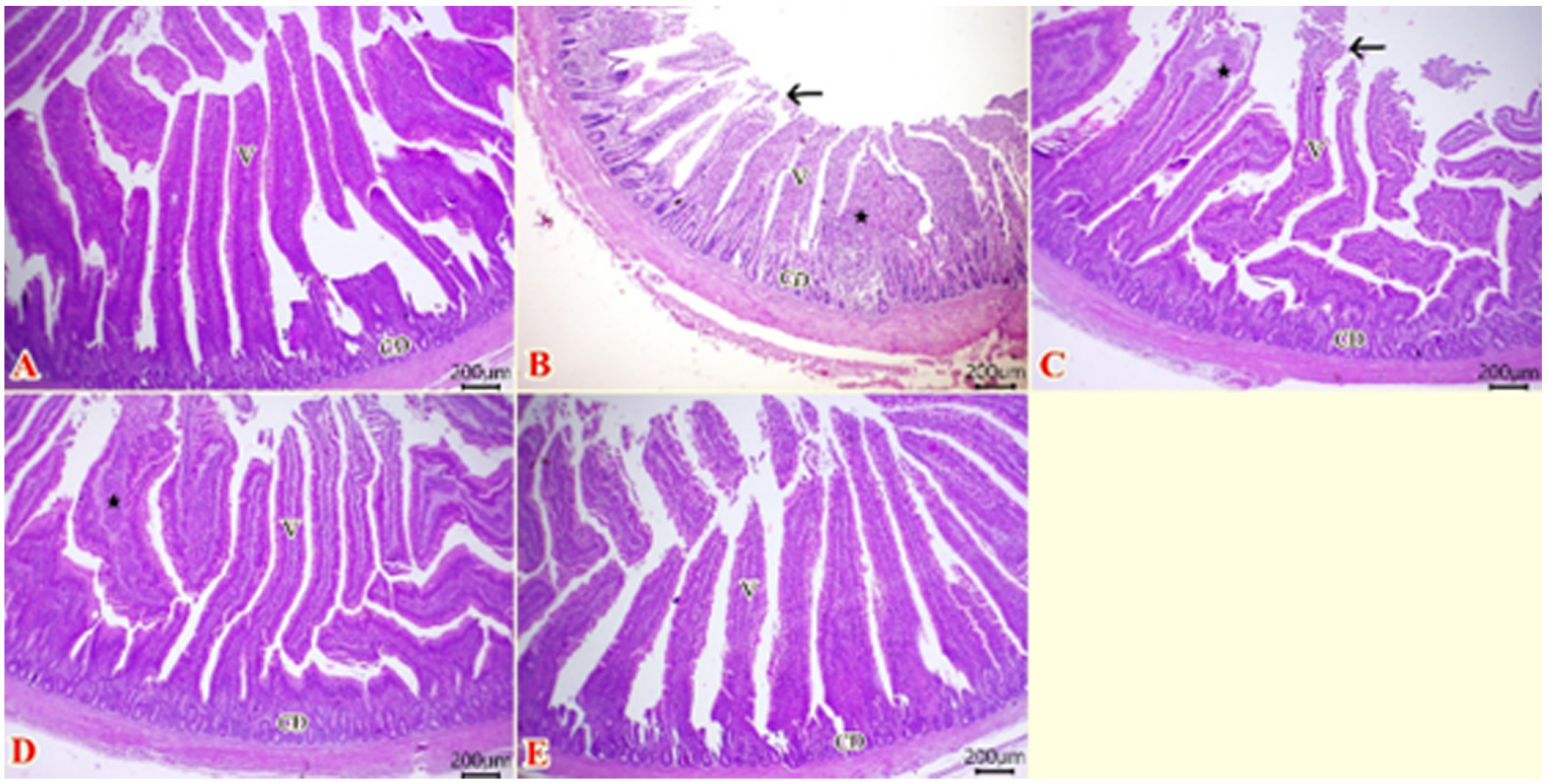
Photomicrograph of H&E-stained intestinal sections (scale bar 200 μm) of broilers offered dietary inclusion with *Salmonella* bacteriophage (BP), grape seed oligomeric pro-anthocyanidins (GSOPs) either individually or in combination, and challenged with *Salmonella* Typhimurium. **(A)** chicks administered a basal diet devoid of any additives and were not subjected to any challenge showed normal histological structures of epithelial lining villi, lamina propria, submucosa, and muscular layer. **(B)** Chicks offered a basal diet without supplements and challenged with *S*. Typhimurium exhibited some necrotic and desquamated intestinal villous tips (arrow), infiltrated lamina propria and submucosa with inflammatory cells (star), and lytic necrosis at the submucosal layer and at some crypts. **(C)** *S*. Typhimurium challenged chicks were offered a basal diet augmented with *Salmonella* bacteriophage (BP) alone at concentrations of 10^9^ PFU/0.1 ml, which revealed some necrotic villous tips (arrow), and some impacted villous cores with inflammatory cells (star). **(D)** *S*. Typhimurium challenged chicks that received a basal diet augmented with grape seed oligomeric pro-anthocyanidins (GSOPs) alone at a level of 400 mg/kg diet showed thickening of some villous cores by inflammatory cells (star) and apparently normal most intestinal layers. **(E)** *S*. Typhimurium challenged chicks were offered a basal diet augmented with a combination of both *Salmonella* BP and GSOPs exhibited ameliorations in the integrity of intestinal layers.

The histopathological analysis of intestinal tissues of broilers offered dietary inclusion with BP, and GSOPs either individually or in combination, and challenged with *S*. Typhimurium at the end of the rearing period, is depicted in Figure 7. The small intestine of negative control broilers showed normal histological architecture of epithelial lining villi, lamina propria, submucosa, and muscular layer (Figure 7A). While the intestinal tissues of *S*. Typhimurium challenged broilers revealed some necrotic and desquamated intestinal villous tips. Lamina propria and submucosa were infiltrated with inflammatory cells with lytic necrosis at the submucosal layer and some crypts (Figure 7B). Post-supplementation with BP, the intestinal histopathological image exhibited enhancements, with some necrotic villous tips, and some villous cores were impacted with inflammatory cells (Figure 7C). Moreover, challenged broilers offered GSOPs exhibited enhancement of the intestinal histological architecture with thickening of some villous cores by inflammatory cells and apparently normal intestinal layers (Figure 7D). These findings were enhanced in GSOPs+BP-fed broilers, where intestinal tissues showed ameliorations in the integrity of intestinal layers with normal histological structures (Figure 7E).

Furthermore, Table 6 illustrates the impact of dietary inclusion with BP, and GSOPs either individually or in combination on intestinal histomorphometry parameters (VL, VW, ASA, and CD). The findings indicated that birds offered dietary BP and GSOPs either individually or in combination substantially (*p* < 0.001) attained the highest VL value in comparison to the IC control, with no significant difference between the treatment groups and the negative control group. Furthermore, VW was considerably higher in GSOPs+BP, GSOPs, and negative control groups (*p* = 0.005) in comparison to the IC group. The maximum significant absorption surface area (ASA) was observed in the GSOPs+BP and negative control groups (0.306 and 0.307 mm^2^, respectively), followed by the GSOPs and BP groups (0.259 and 0.193 mm^2^, respectively), when compared with the IC group (0.104 mm^2^).

**Table 6:** The intestinal histomorphometry of broilers fortified with dietary *Salmonella* bacteriophage (BP), grape seed oligomeric pro-anthocyanidins (GSOPs) either individually or in combination, and challenged with *Salmonella* Typhimurium.

**Intestinal measurements**	**Experimental group**					***p*-value**
	**C**	**IC**	**BP**	**GSOPs**	**BP**+**GSOPs**	
VL, μm	1,760.1 ± 110.7^a^	822.9 ± 36.2^b^	1,434 ± 101.9^a^	1,601.8 ± 77.4^a^	1,714.4 ± 98.1^a^	< 0.0001
VW, μm	176.09 ± 15.3^a^	109.9 ± 10.5^b^	134.8 ± 7.3^ab^	161.26 ± 6.5^a^	178.9 ± 11.9^a^	0.005
ASA, mm^2^	0.307 ± 0.015^a^	0.104 ± 0.014^c^	0.193 ± 0.017^b^	0.259 ± 0.022^ab^	0.306 ± 0.018^a^	< 0.0001
CD, μm	176.8 ± 11.2	160.9 ± 7.5	163.6 ± 29.3	173.3 ± 9.8	181.9 ± 11.6	0.865

VL, villous length, VW, villous width, ASA, absorption surface area, CD, crypt depth.

Results are displayed as means ± SEM (standard error of the mean).

^a − c^Mean values denoted by various letters in the same row exhibit significant differences at *p* < 0.05. **C** (negative control): chicks were administered a basal diet devoid of any additives and were not subjected to any challenge, IC (infective control): chicks were offered a basal diet without supplements and were challenged with S. Typhimurium, BP: chicks were offered a basal diet augmented with Salmonella bacteriophage (BP) alone at concentrations of 10^9^ PFU/0.1 ml, GSOPs: chicks were offered a basal diet augmented with grape seed oligomeric pro-anthocyanidins (GSOPs) alone at a level of 400 mg/kg diet, and GSOPs+BP: chicks were offered a basal diet augmented with combination of both Salmonella BP and GSOPs. The IC and three treatment groups were challenged at 14 days of age with MDR S. Typhimurium strain.

## 4 Discussion

*Salmonella* Typhimurium infection, particularly the MDR strain, has resulted in significant detriment to the poultry sector because of elevated death rates and diminished growth in broilers, in addition to its association with human foodborne illness (22). Therefore, it is essential to implement effective strategies for controlling and reducing salmonellosis in the chicken industry, such as bacteriophages (25) and phytochemicals (26) as alternatives to antibiotics (28). Previous studies successfully utilized *Salmonella* BP (22, 25, 33) and anthocyanins (26, 57) to reduce *S*. Typhimurium in infected broilers and enhance the production performance of broilers. Nevertheless, despite the promising results of individual applications of the two methods to combat *S*. Typhimurium, the synergistic impact of *Salmonella* BP and PAs has not yet been explored for mitigating *S*. Typhimurium infection in broilers. Pondering this situation, the current investigation was designed to explore, for the first time, the combined impacts of dietary *Salmonella* BP and GSOPs on growth performance, immune functions, antioxidant potential, cecal microbiota, gut health, in addition to *S*. Typhimurium resistance in challenged broilers. In this experiment, birds received dietary GSOPs, and GSOPs+BP inclusion exhibited maximum BWG and superior FCR throughout the starter phase, in contrast to the control groups, with no significant difference between the two treatments. The dietary incorporation of BP and GSOPs alone or in combination significantly (*p* < 0.01) contributed to the augmentation of BWG and the reduction of FCR throughout the grower phase in broilers relative to the IC group. The FCR was substantially decreased (*p* < 0.01) in GSOPs+BP than in BP, and in GSOPs-supplemented groups during the finisher period, unlike the infective control group. During the finisher phase, broilers in GSOPs and BP groups showed a substantial (*p* < 0.01) elevation in the BWG, unlike the IC group. Throughout the overall rearing period of 36 days, the impaired BWG, FCR, and elevated mortality percentages observed in groups challenged with *S*. Typhimurium were significantly enhanced in those offered a combination of GSOPs and BP, followed by the GSOPs and BP groups, which suggests the synergistic impact between BP and GSOPs. Accordingly, a recent work stated that dietary BP supplementation significantly boosted BWG, reduced FCR, and mortality percentages in *S*. Typhimurium challenged broiler chickens (25). In accordance, a recent study stated that dietary BP significantly enhanced mortality percentages in *S*. Typhimurium challenged chickens, but could not alleviate the compromised BWG (22). Consistent with our findings, prior research indicated that dietary BP improved FCR and increased BW in *S*. Enteritidis challenged chickens relative to the control group (74). These findings may be ascribed to the antibacterial and immunostimulant characteristics of BP, in addition to its ability to normalize the abnormal intestinal microbial populations caused by *Salmonella* infection (22, 75). Similarly, dietary inclusion of bilberry anthocyanin (26, 57) and protocatechuic acid (76) significantly enhanced growth performance attributes in *S*. Typhimurium challenged broilers. Similarly, previous research reported that dietary GSPOs enhanced the growth performance metrics in chickens (51, 53, 56), but this is the first report of the effect of GSOPEs on the performance of *S*. Typhimurium challenged broilers. The growth-enhancing impacts of GSOPs can be ascribed to their polyphenols, which are considered powerful natural antioxidants with free radical scavenging capacity, and have the ability to enhance the gut microbiota and gut integrity, alongside their anti-inflammatory, immunostimulant, and antibacterial properties (26, 51, 53, 57). Nevertheless, despite the encouraging outcomes of individual applications of BP and PAs to mitigate *S*. Typhimurium, the synergistic use of *Salmonella* BP and GSOPs on the growth performance of *S*. Typhimurium challenged broilers has not yet been explored. Consequently, it has been proposed that a combination of multiple approaches may prove advantageous in controlling bacterial infections in broilers as diverse mechanisms of action could yield additive synergistic impacts throughout the meat production chain, hence enhancing the safety of the end product (24). In this context, our findings indicate a synergistic effect between BP and GSOPs in improving the growth performance metrics of *S*. Typhimurium challenged broilers over the whole growing period.

There is a significant correlation between avian immunity, overall health, and their antioxidant competence. Bacterial infections in chickens disrupt immunity, antioxidant capacity, and the synthesis of reactive oxygen and nitrogen species, which results in physiological alterations linked to oxidative stress (1, 19). The excessive generation of reactive oxygen species can harm tissues, provoke lipid peroxidation, and disrupt cellular biological functions, thus diminishing avian performance, wellbeing, and survival, ultimately resulting in economic losses (77–79). Conversely, elevated amounts of free radicals provoke lipid peroxidation, leading to oxidative damage and elevated MDA levels, which contribute to post-mortem meat degradation (80, 81). The removal of excessive ROS by robust endogenous antioxidant defense systems, including GSH-Px, SOD, and CAT enzymes, maintains cellular homeostasis and safeguards against oxidative damage (42). Thus, providing chickens with natural feed additives, particularly phytochemicals possessing immunostimulant qualities, may enhance their antioxidant response by eliminating free radicals and mitigating the detrimental effects of ROS, thereby improving meat quality and extending its shelf life post-slaughter (82, 83). In this context, GSOP is a naturally occurring antioxidant; nevertheless, further investigation is required to elucidate its impact on the antioxidant response in broiler chickens and to determine if its application could confer supplementary advantages for enhancing this function. We demonstrated a substantial rise in the concentrations of GSH-Px, SOD, and CAT enzymes, alongside a notable reduction in the levels of H_2_O_2_, ROS, and MDA, at 7 dpc, in the intestinal tissues of *S*. Typhimurium challenged broilers following dietary fortification with GSOPs, BP, and their combination, compared to the IC group. Dietary fortification with BP, GSOPs, and their combination, particularly GSOPs+BP, mitigated the negative impact of *S*. Typhimurium infection on the oxidative and antioxidant attributes, and restored their activities to levels comparable to those in the negative control group, which indicates their role in activating the antioxidant response, thereby improving the avian immune system and overall health. In accordance, broilers administered diets supplemented with BP exhibited increased levels of SOD, GSH-Px, and reduced MDA concentrations in *S*. Enteritidis challenged broilers (64). Accordingly, a previous study showed that dietary GSOPs inclusion significantly reduced MDA levels, and increased the concentrations of CAT and GSH-Px antioxidant enzymes in chickens exposed to aflatoxin B1, unlike the control positive group (51). Similarly, dietary GSOPs supplementation significantly increased SOD level and decreased MDA concentrations in *Eimeria*-challenged broilers, unlike the IC group (53). Similar to this, dietary GSOPs inclusion significantly elevated SOD concentrations and reduced MDA levels in broilers relative to the control group (56). Similarly, a previous work stated that dietary GSOPs significantly increased SOD, CAT, and GSH-Px concentrations and reduced MDA levels in fish (84). However, to date, there has been no investigation on the antioxidant capacity of dietary GSOPs+BP in *S*. Typhimurium challenged broilers.

Renal and hepatic functions can be evaluated by analyzing irregular variations in the serum levels of urea, creatinine, AST, and ALT (1, 85). Herein, following the *S*. Typhimurium challenge, hepatic and renal functions exhibited reduced serum amounts of ALT, AST, creatinine, and urea, potentially attributable to the detrimental impact of bacterial infection on hepatorenal tissues. Dietary inclusion with BP, GSOPs, and their combination, particularly GSOPs+BP, demonstrated a progressive increase of these parameters toward normal values comparable to the negative control group, thus indicating its hepato-renal protective properties. Accordingly, recent work depicted that dietary BP supplementation significantly reduced the elevated AST and ALT levels in *S*. Typhimurium (86) and *S*. Enteritidis (31) challenged broilers, unlike the control positive group. Similarly, a previous study stated that dietary GSOPs inclusion significantly reduced chickens' serum AST and ALT levels, following aflatoxin B1 challenge, toward normal values relative to the control negative group, which suggests a protective effect on the broilers' hepatic tissues (51). In accordance, previous studies reported that dietary GSOPs significantly reduced serum levels of AST and ALT in fish (84) and rats (87). Similarly, broiler serum concentrations of uric acid, creatinine, ALT, and AST were significantly reduced in response to dietary phytochemicals inclusion in heat-stressed broilers, unlike the control positive group (88). Nonetheless, the combined impact of *Salmonella* BP and GSOPs on the liver and kidney function profiles of *S*. Typhimurium challenged broilers has not yet been investigated.

Serum immunological-related indices are critical indicators that offer significant knowledge regarding the general health of broilers, since their immune system predominantly governs their wellbeing (37). Bacterial infections induce systemic inflammatory responses, posing a significant challenge to immune function, jeopardizing health, and leading to diminished avian performance. Elevated levels of LYZ and NO, which are primarily produced by phagocytic cells, may indicate a response to bacterial infection and serve as critical markers of inflammation (2, 89). C-reactive protein is produced at sites of infection or inflammation by various cells, such as endothelial cells, macrophages, and lymphocytes, and it is regarded as an acute inflammatory protein that mitigates inflammation, making it a potential marker for decreased tissue damage and body inflammatory responses (90). Moreover, CRP is crucial for the synthesis of cytokines, in addition to facilitating phagocytosis and nitric oxide production in response to bacterial infection (2, 91). IgM and IgG are two of the three principal immunoglobulin isotypes that react to both local and systemic infections (92). Immunoglobulins are essential components of humoral immunity, as they significantly contribute to immune defense mechanisms, including phagocytosis and the neutralization of harmful germs (42). Complement is a glycoprotein enzyme that functions in immune regulation. Furthermore, immunoglobulin may elevate complements C3 and C4, so enhancing the liver's defenses against infections and fortifying the immune response (1, 93). Simultaneously, our outcomes revealed a reduction in serum LYZ, NO, CRP, and complement C3 levels, along with an elevation in serum levels of IgG and IgM at 1 week post-challenge with *S*. Typhimurium in broilers receiving dietary BP, GSOPs, and their combination, demonstrating their potential efficacy in mitigating the detrimental impact of *S*. Typhimurium infection. At 7 dpc, birds offered dietary BP, and GSOPs combination exhibited the most significant (*p* < 0.01) immunological reaction, as indicated by reduction in the serum concentrations of LYZ, NO, CRP, and complement C3, alongside increased serum IgG level, unlike the IC group, which suggests synergistic positive impact between BP and GSOPs. This may pertain to the immunostimulant and anti-inflammatory properties of the dietary BP and GSOPs combination. Accordingly, a recent work showed that dietary BP fortification significantly increased serum levels of IgG and IgM in *S*. Enteritidis challenged broilers, unlike the control positive group (86). In accordance, a recent work reported that dietary BP supplementation significantly reduced the elevated CRP levels in *S*. Typhimurium challenged broilers, unlike the control positive group (86), which suggests the anti-inflammatory activity of *Salmonella* BP. Similarly, dietary bilberry anthocyanin reduced the elevated serum NO level in *S*. Typhimurium challenged broilers, unlike the control positive group (26). In the same line, dietary GSOPs supplementation reduced the elevated serum NO level in *Eimeria*-challenged broilers (53). Accordingly, dietary GSOPs inclusion significantly boosted serum IgG and IgM activities in broilers exposed to aflatoxin B1 (51); nonetheless, the immunostimulant and anti-inflammatory properties of the dietary BP and GSOPs combination in *S*. Typhimurium challenged broilers have yet to be investigated.

The gastrointestinal mucosa serves as the primary location for nutritional digestion and absorption, while also playing a vital role in safeguarding the host against infections and preventing the translocation of proinflammatory chemicals into the bloodstream (1, 94). The gastrointestinal mucosa integrity is upheld by TJP (JAM-2, claudins-1, and occludin) between neighboring epithelial cells and enterocytes, which are essential for the formation of an integral physical barrier across cells in the gut epithelium (2, 95). The impairment of TJPs is a significant contributor to “leaky gut,” which may result in diminished nutritional absorption, increased luminal antigens permeability, translocation of pathogens, tissue injury, and persistent inflammatory conditions (37, 96). Research has shown that dietary phytochemicals and BP inclusion may improve gut barrier integrity by facilitating the synthesis of TJP (97). In addition to TJP, the mucus layer serves as the primary protective barrier against enteric pathogens, with mucins as its principal constituents (98). Mucins, particularly the *Muc-2* gene, which is expressed by intestinal goblet cells, serve as the primary barrier of immunological defense, and enhancing their secretion is beneficial in preventing bacterial invasion and toxin dissemination throughout the gastrointestinal tract (85). Consequently, infections with enteric pathogens in broilers have been demonstrated in many studies to result in the minimizing the expression of *Muc-2* gene (1, 63), which is consistent with our findings, where *S*. Typhimurium infection compromises the intestinal barrier functions of challenged broilers via downregulating *JAM-2*, CLDN-1, occludin, *MUC-2*, β-defensin-1, and cathelicidins-2 genes, subsequently detrimentally impacting their BWG and FCR. In addition to *MUC-2* protective characteristics, it plays a role in nutrient filtration inside the gastrointestinal tract and can influence the digestion and absorption of nutrients, which may account for the enhanced growth performance of broilers observed in the present research. Notably, host defense peptides (HDP) are broad-spectrum antimicrobial compounds generated by the gastrointestinal mucus layer, contributing to the gut innate immune system and mucosal barrier, with their gene transcription levels influenced by bacterial regulation (89, 99). Furthermore, previous research has shown that HDP directly regulates the expression of the TJPs and mucin genes, which improves mucosal barrier permeability (100). The incorporation of phytochemicals and BP in broilers' nutrition and the evaluation of their effects on the gut integrity of broilers are emerging topics that necessitate more research to elucidate their mechanisms of action; however, the preventive effects of GSOPs and BP combination in safeguarding broilers from *S*. Typhimurium infection have not been examined to date. Consistent with the aforementioned facts, our findings revealed that alongside the enhanced growth performance in broilers receiving dietary BP, GSOPs, and their combination, the transcription levels of genes encoding TJP [*JAM-2, CLDN-1*, and occludin], mucins and [*MUC-2*], and HDP [β-defensin-1, and cathelicidins-2] were similarly elevated, particularly GSOPs+BP group, at 7 dpc with *S*. Typhimurium, underscoring their efficacy in enhancing intestinal barriers. The enhancing effect of dietary GSOPs' inclusion on the transcription of genes encoding intestinal barriers could be related to their principal bioactive substances. In agreement with our outcomes, dietary *Salmonella* BP upregulated the transcript levels of genes related to TJP (occludin and ZO-1) in broilers (97). Similarly, dietary BP inclusion elevated the transcript levels of genes related to TJP (ZO-1, *CLDN-1*, and occludin) in the jejunum of piglets (101). Accordingly, dietary bilberry anthocyanin upregulated the transcript levels of genes related to mucins (*MUC2)* (26, 57), and TJP (occludin, *CLDN-1*, and *ZO-1*) (26) in *S*. Typhimurium challenged broilers, unlike the control positive group. In accordance, dietary GSOPs supplementation elevated the transcript levels of *MUC-2* and occludin genes in pigs (102); however, the efficacy of dietary GSOPs and BP combination on the expression of genes encoding intestinal barriers has not been investigated yet.

Autophagy is an essential system that maintains cellular homeostasis and physiological functions, such as immune defense, development, and reproduction (103). Furthermore, it functions as a cellular defense system against external deleterious stimuli by degrading impaired organelles, protein clumps, and microorganisms within cells by lysosomes (19). The onset of autophagy relies on the involvement of certain autophagy-related (atg) genes, including *atg5, atg7, atg12, bclN-1, LC3-II*, and *mTOR* (1). The atg5 protein is crucial for the formation of autophagic vacuoles and is highly conserved among most eukaryotes (104). The *BCLN-1* gene, which is thought to be a homolog of yeast *atg6*, is a target gene linked to autophagy in human cells and is involved in the creation of autophagosomes. Moreover, *BCLN-1* plays a crucial role in tumor growth by regulating autophagic activity [89]. *LC3-II* is regarded as an autophagic marker since its amount is correlated with the quantity of autophagic vacuoles (105). Moreover, the autophagy process and cellular metabolism are significantly influenced by the *mTOR* gene, and there is an inverse relationship between *mTOR* activation and autophagy commencement (106). Herein, the inclusion of BP, and GSOPs combination in broilers diet significantly (*p* < 0.05) downregulated *mTOR* expression, alongside upregulating the transcriptional levels of *BCLN-1, LC3-II, atg5, atg7*, and *atg12* genes in the cecal tissues of challenged broilers at 7 dpc with *S*. Typhimurium strain, in comparison to the IC group, which indicate faster clearance of *S*. Typhimurium. In accordance, prior studies indicated that dietary phytochemicals inclusion enhanced the expression of *BCLN-1, LC3-II, atg5, atg7*, and *atg12* genes while suppressing the *mTOR* gene in broilers (1, 103); nevertheless, no research has examined the impact of dietary BP and GSOPs combination on autophagy-related genes in *S*. Typhimurium challenged broilers.

The gastrointestinal microbial populations play a vital function in maintaining the integrity of the gastrointestinal mucosa (2, 107). The ability of phytochemicals and BP to combat harmful and unfavorable bacteria in the gastrointestinal system is one of their main and most important biological functions (26, 74). The relationship between nutrient utilization and the gut microbiota of broilers has been the focus of recent investigations (108). This is an essential mechanism for the growth-promoting properties of antimicrobial agents and a framework for creating alternatives via microbiota manipulation to improve the wellbeing and performance of broilers (109). The outcomes of this experiment revealed that GSOPs+BP supplementation altered the cecal microbiota composition in favor of beneficial bacteria compared to the IC group at 7 dpc. At 7 dpc with *S*. Typhimurium, dietary fortification with BP, and GSOPs combination significantly reduced the counts of cecal pathogenic bacteria, including *Enterobacteriaceae, Escherichia, Clostridium* clusters I and IV, while increasing the load of cecal beneficial bacteria, including *Bacteroides, Firmicutes, Bifidobacterium*, and *Lactobacillus* spp., when compared with the IC group. In agreement with our findings, dietary BP increased the count of beneficial bacteria, including *Lactobacillus* spp., in *S*. Enteritidis challenged broilers (64, 74). Similarly, dietary BP increased the load of beneficial bacteria, including *Lactobacillus* spp. (22), while reducing the pathogenic bacteria loads, including *Clostridium* (22), and coliform (31) in *S*. Typhimurium challenged broilers. Accordingly, protocatechuic acid significantly increased the abundance of *Lactobacillus* and *Firmicutes*, while reducing *Escherichia* count in *S*. Typhimurium challenged broilers (76). In the same line, dietary anthocyanins significantly decreased the abundance of pathogenic bacteria, including *Proteobacteria*, while increasing the abundance of *Firmicutes* in *S*. Enteritidis challenged broilers (26). Similar to this, dietary GSOPs significantly elevated the count of the *Firmicutes* bacteria and reduced the pathogenic bacteria, such as *Proteobacteria* and *Cyanobacteria*, in fish (110). These results are linked to the ability of phytochemicals and BP to maintain the normal gut microbiota abundance via enhancing the growth and metabolic function of beneficial bacteria, while diminishing the count and metabolic function of the harmful ones, which in turn positively influence the avian wellbeing and performance (26, 74).

The growth of enteric bacteria often leads to chronic inflammation reactions that diminish chicken productivity and increase the risk of contamination of chicken products (111). Infection with *S*. Typhimurium in chickens has been shown to elevate death rates and induce gastrointestinal lesions, which are linked to human foodborne illnesses (112). The quantitative analytical outcomes of *S*. Typhimurium in the cecal contents following the challenge demonstrated that the addition of dietary BP, and GSOPs combination consistently reduced *S*. Typhimurium counts in the cecal contents of challenged broilers at 7, and 14-pdc with MDR *S*. Typhimurium strain compared to the IC group, which indicate their antibacterial properties, and a possible synergistic impact between BP, and GSOPs. Our results support the findings of other research, where dietary BP inclusion significantly reduced *S*. Typhimurium (22, 25) and *S*. Enteritidis (31, 74) abundance in challenged broilers, which provided some protection for chickens against *Salmonella* infection and mitigated further mortalities. Accordingly, dietary anthocyanins (26) and protocatechuic acid (76) inclusion decreased *S*. Typhimurium counts in challenged chickens; nonetheless, the impact of dietary GSOPs+BP incorporation on the cecal *S*. Typhimurium populations in challenged broilers has yet to be examined. These results may be ascribed to cell wall disintegration, membrane protein, and cytoplasmic membrane damage, reduction of the proton motive force, cytoplasmic coagulation, and cellular elements leakage. Furthermore, substantial histological changes, including degenerative alterations and significant leukocytic infiltration, along with a damaged mucosal barrier, were observed in the intestinal tissues of broilers infected with S. Typhimurium (infective control group). Similar outcomes were formerly noted in the intestinal tissues of broilers challenged with *S*. Typhimurium (26, 76). Dietary supplementation with BP and GSOPs combination significantly impeded the transfer of *S*. Typhimurium to other organs, as evidenced by the restoration of the normal intestinal histopathological structure in broilers, indicating its beneficial effect on avian immune systems, health, and wellbeing. Similarly, dietary inclusion with dietary anthocyanins (26) and protocatechuic acid (76) leads to substantial improvement in the intestinal histological structure of *S*. Typhimurium challenged broilers. In accordance, the administration of dietary BP to broilers challenged with *S*. Enteritidis (31, 74) resulted in significant enhancements in the intestinal histological structure. This may pertain to the advantageous effects of GSOPs+BP, as evidenced by our data, in enhancing broilers' immunity against enteric bacterial infections and strengthening intestinal barriers, hence preventing the dissemination of infections to other organs. Moreover, regarding *S*. Typhimurium resistance in challenged birds after dietary GSOPs+BP inclusion, prior studies indicated that phytochemicals and BP can change the innate immune reaction by diminishing bacterial survival, augmenting NO generation, and enhancing macrophage phagocytic capacity (2, 31).

Nutritional immunology is an innovative approach for controlling bacterial illnesses in the broiler sector, bypassing the limitations of immunization strategies through the utilization of feed supplements (43). Moreover, enhancing the nutrition and medical treatment of broilers will render their rearing more economical and effective by reducing infections; thus, numerous treatment strategies currently emphasize bacterial pathogenicity rather than bacterial survival (2). The pathogenicity of *Salmonella* depends on numerous virulence attributes and manifests itself in three ways: invasion, survival intracellularly, and colonization. *Salmonella* employs virulence markers to penetrate the epithelial cells of the gut and persist inside mucosal macrophages, resulting in an acute inflammatory reaction (63). Hence, the anti-virulence characteristics of GSOPs, BP, and their combination were assessed by measuring the transcript levels of the *S*. Typhimurium *sopE* and *spvC* virulence genes following their supplementation at 7 dpc with the MDR *S*. Typhimurium strain. Our results indicated that the most pronounced downregulation in *S*. Typhimurium *sopE* and *spvC* transcript levels was observed in the cecal tissues of GSOPs+BP, followed by BP, then GSOPs fed birds, when compared with the IC group at 7 dpc, which suggests a potential synergistic effect between BP and GSOPs. Accordingly, prior investigations reported the *in vitro* antivirulence properties of BP (113, 114) and PAs (115–117) against *S*. Typhimurium virulence genes. In accordance, prior studies reported the *in vivo* anti-virulence activity of nanoparticles loaded natural products against *Mycoplasma gallisepticum* and/or *Ornithobacterium rhinotracheale* in challenged broilers (118). Similarly, previous research demonstrated the *in vivo* anti-virulence efficacy of phytochemicals against *C. jejuni* (43) in challenged broilers, but the *in vivo* anti-virulence impact of GSOPs and BP combination in *S*. Typhimurium challenged broilers has yet to be investigated.

Cytokines are essential regulators of the gastrointestinal inflammatory responses and play a significant role in the immune response against bacterial infections. Gastrointestinal immunological cells are stimulated to secrete cytokines upon the invasion of microbes into the gut epithelium (2, 43). Proinflammatory cytokines such as *IL-1*β and *IL-6* are integral to acute-phase inflammation that is linked to both systemic and metabolic alterations (119), while also modulating the immune response during bacterial infections (120). Gastrointestinal infections may upregulate *IL-6* and *IL-1*β genes, therefore enhancing the permeability of the gastrointestinal epithelial cells (121). Chemotactic cytokines, also known as chemokines, are inflammatory proteins produced by macrophages, such as CCL4 and CCL20, and are fundamental for controlling the host's immunological reaction to illness (2). As a chemoattractant, *CCL4* attracts important immunological cells such as natural killer cells, T lymphocytes, dendritic cells, monocytes, and macrophages (122). Moreover, the production of *CCL4* and *CCL20* by neutrophils facilitates inflammation by recruiting extra leukocytes to the site of inflammation, subsequently prompting macrophage-mediated resolution of inflammation responses and leading to chronic inflammatory conditions (89, 123). Furthermore, the physiologic conversion of arachidonic acid into inflammatory prostaglandins, which in turn initiates the synthesis of cytokines, is mediated by the COX-2 enzyme (1, 124). Significantly, pro-inflammatory mediators such as COX-2 and iNOS mutually regulate the inflammatory process induced by bacterial invasion (2, 89). Phytochemicals exhibit anti-inflammatory and immunostimulant characteristics in various avian immunologic and inflammatory disorders, demonstrating ameliorating impacts on proinflammatory cytokines such as *IL-6* and *IL-1*β, thereby mitigating intestinal inflammation and maintaining gastrointestinal homeostasis (103). Herein, in conjunction with enhancing the serum immunological attributes, dietary fortification with BP, GSOPs, or their combination significantly minimized the heightened inflammatory response subsequent to *S. Typhimurium* challenge. At 7 dpc with S. Typhimurium, incorporation of dietary BP and GSOPs combination significantly downregulated the mRNA transcript levels of genes encoding proinflammatory cytokine (*IL-6* and *IL-1*β), chemokines (*CCL4* and *CCL20*), alongside *COX-2* and *iNOS* genes, concerning the IC group, demonstrating their significant immunostimulant and anti-inflammatory effects (125). Similarly, dietary BP fortification significantly minimized the transcript levels of *IL-1*β and *IL-6* genes in *S*. Typhimurium (126, 127) and *S*. Enteritidis (128) challenged chickens. In agreement with our findings, dietary anthocyanins significantly reduced the transcriptional levels of *IL-1*β and *IL-6* genes following *S*. Typhimurium challenge in broilers (26, 57). In a comparable work, dietary GSOPs inclusion significantly reduced the transcriptional level of *IL-1*β and *IL-6* genes in broilers exposed to aflatoxin B1 (51). Similar to this, dietary PAs significantly reduced the transcription level of *IL-6* (54, 55) and *IL-1*β genes, along with significantly decreasing iNOS activity in chickens (55). Similarly, a recent work reported that dietary GSOPs inclusion markedly downregulated *CCL4, IL-1*β, and *IL-6* genes in rats. In the same line, dietary phytochemical inclusion significantly decreased the transcriptional levels of *IL-1*β*, IL-6, CCL4, CCL20, COX-2*, and *iNOS* genes in broilers (1, 2, 89). Nevertheless, the influence of dietary GSOPs and BP combination on genes associated with cytokines and chemokines in *S*. Typhimurium challenged broilers has yet to be investigated. In our assessment, dietary GSOPs and BP combination has immunostimulant, anti-inflammatory, antibacterial, and anti-virulence properties that enhance cellular and humoral immune systems, hence reducing the development of pathogenic bacteria and inflammatory responses in broiler chickens.

## 5 Conclusion

Our outcomes concluded that the incorporation of *Salmonella* BP and GSOPs, or their combination, particularly GSOPs+BP, serves as a potential feed additive for broiler diets, in accordance with the worldwide objective of prohibiting the indiscriminate use of antimicrobials while promoting efficient and sustainable chicken production. The dietary BP, GSOPs, and their combination enhanced the intestinal barriers and TJP, hence optimizing performance in *S*. Typhimurium challenged broilers. The correlation between decreased *S*. Typhimurium loads and its virulence indicates the direct antimicrobial efficacy of dietary BP, GSOPs, and their combination, especially GSOPs+BP, in enhancing the broiler gastrointestinal microenvironment, barrier functions, inflammatory response, and immune systems, thus impeding *S*. Typhimurium colonization and its impact on gastrointestinal mucosal damage. Considering the practical implications of these findings, the GSOPs and *Salmonella* BP combination can serve as a reliable substitute feed additive to antimicrobial growth boosters in managing economically important gastrointestinal illnesses, including salmonellosis.

## Data Availability

The data displayed in this study is available on request from the corresponding author.
