# Supplementary material for: Novel integrated approach modeling proanthocyanidins and bacteriophages to combat multidrug Salmonella Typhimurium in challenged broilers
Source: Front Vet Sci. 2025 Nov 26;12:1694544. doi: 10.3389/fvets.2025.1694544 (PMC12690214; doi:10.3389/fvets.2025.1694544)
Supplement: Supplementary file 1 [file Table_1.docx]

**Supplementary Table S1.** Primers' sequences employed for qPCR assay

| **Accession No./ reference** | **Standard curve validation** | **Primer sequence (5'-3')** | **Specificity/ Target gene** |
| --- | --- | --- | --- |
|  |  | ***Salmonella* Typhimurium** | |
| KF026356 | R^2^= 0.998 E%= 99.5 LOD= –2.2 | F-GTGAAATTATCGCCACGTTCGGGCAA | *invA* |
|  |  | R- TCATCGCACCGTCAAAGGAACC |  |
|  |  |  | **Cecal microbiota** |
| (1) |  | F-GTTAATACCTTTGCTCATTGA | *Escherichia* |
|  | R^2^= 0.997 E%= 106.5 LOD= –2.04 | R-ACCAGGGTATCTAATCCTGT |  |
|  |  | F-CATTGACGTTACCCGCAGAAGAAGC | *Enterobacteriaceae* |
| (2) | R^2^=0.995 E%= 96.34 LOD= –2.19 | R-CTCTACGAGACTCAAGCTTGC |  |
|  |  | F-GGAGYATGTGGTTTAATTCGAAGCA | *Firmicutes* |
|  | R^2^=0.998 E%= 99.07 LOD= –2.15 | R-AGCTGACGACAACCATGCAC |  |
|  |  | F-GGARCATGTGGTTTAATTCGATGAT | *Bacteroidetes* |
|  | R^2^=0.998 E%= 90.99 LOD= –2.29 | R-AGCTGACGACAACCATGCAG |  |
|  |  | F-TCGCGTCYGGTGTGAAAG | *Bifidobacterium*  species |
|  | R^2^=0.998 E%= 92.75 LOD= –2.26 | R-CCACATCCAGCRTCCAC |  |
|  |  | F-AGCAGTAGGGAATCTTCCA | *Lactobacillus* species |
|  | R^2^=0.999 E%= 93.33 LOD= –2.25 | R-CACCGCTACACATGGAG |  |
|  |  | F-GCACAAGCAGTGGAGT | *Clostridium* cluster  IV |
|  | R^2^ = 0.992 E%= 93.73 LOD= –2.24 | R-CTTCCTCCGTTTTGTCAA |  |
|  |  | F-TACCHRAGGAGGAAGCCAC | *Clostridium* cluster I |
| (3) | R^2^ = 0.999 E%= 96.5 LOD= –2 | R-GTTCTTCCTAATCTCTACGCAT |  |

*invA: S.* Typhimurium invasion protein A gene. “X” is representative the value of “Cycle threshold; Ct”of PCR, and“y” is representative the Log_10_ DNA gene copies quantification data. E%: Efficiency percentage, LOD: limit of detection.

1. Yuan Y, Xu W, Luo Y, Liu H, Lu J, Su C, Huang K. Effects of genetically modified T2A-1 rice on faecal microflora of rats during 90 day supplementation. *J Sci Food Agric* (2011) 91:2066–2072. doi: 10.1002/JSFA.4421

2. Sun H, Ni X, Song X, Wen B, Zhou Y, Zou F, Yang M, Peng Z, Zhu H, Zeng Y, et al. Fermented Yupingfeng polysaccharides enhance immunity by improving the foregut microflora and intestinal barrier in weaning rex rabbits. *Appl Microbiol Biotechnol* (2016) 100:8105–8120. doi: 10.1007/S00253-016-7619-0/FIGURES/7

3. Song Y, Liu C, Finegold SM. Real-Time PCR Quantitation of Clostridia in Feces of Autistic Children. *Appl Environ Microbiol* (2004) 70:6459–6465. doi: 10.1128/AEM.70.11.6459-6465.2004
